# Kras^G12D^ induces changes in chromatin territories that differentially impact early nuclear reprogramming in pancreatic cells

**DOI:** 10.1186/s13059-021-02498-6

**Published:** 2021-10-14

**Authors:** Angela J. Mathison, Romica Kerketta, Thiago Milech de Assuncao, Elise Leverence, Atefeh Zeighami, Guillermo Urrutia, Timothy J. Stodola, Marina Pasca di Magliano, Juan L. Iovanna, Michael T. Zimmermann, Gwen Lomberk, Raul Urrutia

**Affiliations:** 1 Genomic Science and Precision Medicine Center (GSPMC), Medical College of Wisconsin, Milwaukee, WI USA; 2 Division of Research, Department of Surgery, Medical College of Wisconsin, Milwaukee, WI USA; 3 Department of Surgery, University of Michigan, Ann Arbor, MI USA; 4 Centre de Recherche en Cancérologie de Marseille (CRCM), INSERM U1068, CNRS UMR 7258, Aix-Marseille Université and Institut Paoli-Calmettes, Parc Scientifique et Technologique de Luminy, Marseille, France; 5 Clinical and Translational Sciences Institute, Medical College of Wisconsin, Milwaukee, WI USA; 6 Department of Pharmacology and Toxicology, Medical College of Wisconsin, Milwaukee, WI USA; 7 Department of Biochemistry, Medical College of Wisconsin, Milwaukee, WI USA

**Keywords:** Pancreatic cancer, KRAS, Epigenomics

## Abstract

**Background:**

Pancreatic ductal adenocarcinoma initiation is most frequently caused by Kras mutations.

**Results:**

Here, we apply biological, biochemical, and network biology methods to validate GEMM-derived cell models using inducible Kras^G12D^ expression. We describe the time-dependent, chromatin remodeling program that impacts function during early oncogenic signaling. We find that the Kras^G12D^-induced transcriptional response is dominated by downregulated expression concordant with layers of epigenetic events. More open chromatin characterizes the ATAC-seq profile associated with a smaller group of upregulated genes and epigenetic marks. RRBS demonstrates that promoter hypermethylation does not account for the silencing of the extensive gene promoter network. Moreover, ChIP-Seq reveals that heterochromatin reorganization plays little role in this early transcriptional program. Notably, both gene activation and silencing primarily depend on the marking of genes with a combination of H3K27ac, H3K4me3, and H3K36me3. Indeed, integrated modeling of all these datasets shows that Kras^G12D^ regulates its transcriptional program primarily through unique super-enhancers and enhancers, and marking specific gene promoters and bodies. We also report chromatin remodeling across genomic areas that, although not contributing directly to cis-gene transcription, are likely important for Kras^G12D^ functions.

**Conclusions:**

In summary, we report a comprehensive, time-dependent, and coordinated early epigenomic program for Kras^G12D^ in pancreatic cells, which is mechanistically relevant to understanding chromatin remodeling events underlying transcriptional outcomes needed for the function of this oncogene.

**Supplementary Information:**

The online version contains supplementary material available at 10.1186/s13059-021-02498-6.

## Background

Epigenomic regulators are emerging as key critical downstream effectors of driver mutations during the process of cancer initiation, progression, metastasis, and tumor heterogeneity [1]. Pathogenic variants in *KRAS* act as driver mutations in many cancers, with pancreatic cancer being the most frequent. In this work, we use a mechanistically oriented, multi-omics approach based on the integrative analyses and modeling of several next-generation sequencing technologies to define epigenomic changes that underlie Kras^G12D^-mediated effects. Using cells derived from genetically engineered mouse models (GEMM) expressing this oncogene in an inducible manner, we systematically describe the impact of Kras^G12D^ on both methylation changes and chromatin remodeling that account for transcriptional and non-transcriptional responses to signaling by this oncogene. First, using ChIP-seq, we demonstrate that while Kras^G12D^ induces changes in heterochromatic and euchromatic histone marks, its effects on gene expression can be primarily explained by remodeling of enhancers and super-enhancers (H3K27ac and H3K4me1), promoters (H3K4me3), and gene body-associated functions (H3K36me3). Integration of all multi-omics data sets show that changes in heterochromatin marks (H3K9me3 and K27me3) have a minor impact on the transcriptome. Our data also provides insight into the repertoire of writer, reader, and eraser proteins, for which expression is consistent with deposition of their target marks. This data confirms and extends previous studies, by integrating more omics methodologies that are concomitantly performed in a tightly controlled and inducible GEMM-derived cell system [2]. Thus, this systematic investigation highlights the ability of Kras^G12D^ to give rise to an epigenomic landscape that is conducive to pancreatic cell growth. Mechanistically, the finding that Kras^G12D^ induced chromatin remodeling, primarily involving similar pathways for both gene activation and repression, helps to focus future experiments aimed at drugging these pathways to antagonize Kras^G12D^ functions. Moreover, we describe chromatin remodeling events that do not appear to be directly associated with transcription, though may still account for Kras^G12D^ effects. Thus, because the pathways studied here have increasingly available druggable options, this result bears biomedical relevance for chemoprevention and therapeutic studies.

## Results

### Cells derived from genetically engineered mouse models recapitulate early oncogenic *Kras*^*G12D*^ signaling in vitro

**Fig. 1:**
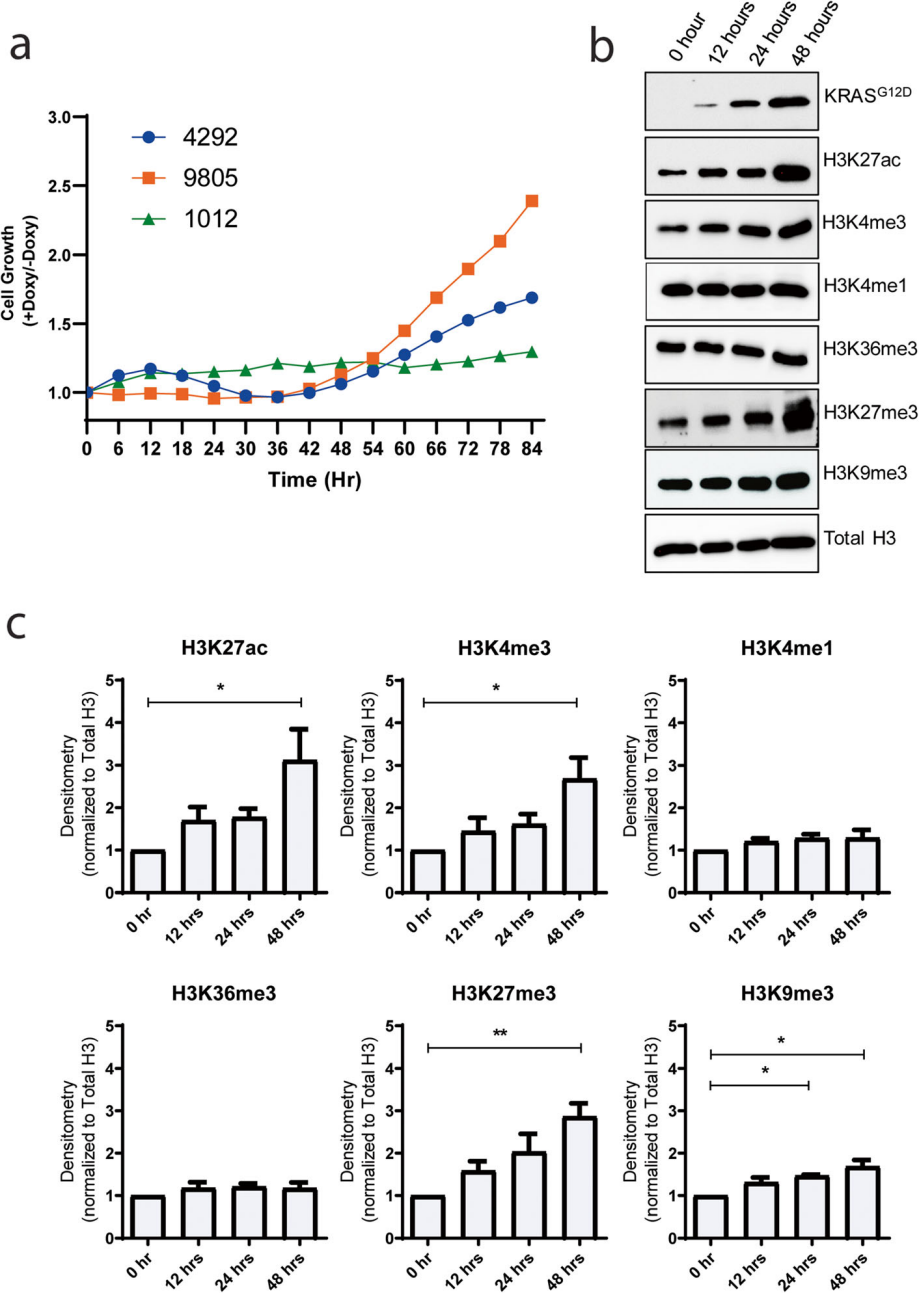
Induction of *Kras*^*G12D*^ leads to changes in cell features. **a** i*Kras* cells (4292, 9805, and 1012) were cultured without doxycycline (0 hr) or treated with doxycycline (48 hrs) to induce *Kras*^*G12D*^. Proliferation was measured by cell confluence in all four lines and expressed as a ratio of *Kras*^*G12D*^-expressing cells to control for each cell line. Cell confluency is an average of three separate experiments for each line. **b** Western blot analysis was performed in 4292 i*Kras* cell line at 0, 12, 24, and 48 hrs time points. Cell lysates were probed with the indicated antibodies for KRAS^G12D^ and the histone marks. **c** Densitometry of western blots was performed on three separate experiments. * and ** indicates *p* value < 0.05 and 0.01 respectively. All data is expressed as mean ± SEM

First, we sought to better understand how Kras^G12D^ coordinates over time, the reorganization of the genome. We used GEMM-derived cell models that carry a doxycycline-inducible *Kras*^*G12D*^ transgene (iKras cell lines designated 4292, 9805, and 1012) [3, 4]. However, the ability of these cells to serve as models for studying molecular signaling and transcription coupling events is not known. Here, we show that induction of the oncogene by doxycycline increases their growth linearly after 48 hours (hrs), which was considered here as early events in Kras^G12D^ function (Fig. 1a). We initiated our studies by using the 4292-cell line, although aspects of the data were also validated in the whole subset of cell lines. Total levels of histone marks were measured by western blot for enhancers (H3K27ac, H3K4me1), super-enhancers (H3K27ac), active promoters (H3K4me1 and H3K4me3), transcriptionally active gene bodies (H3K36me3) and silent heterochromatin (H3K27me3 and H3K9me3). We observed significant (*p* value < 0.05) increases in the levels of enhancers, super-enhancers, and active promoters through the H3K27ac (3.1 ± 0.7 fold) and H3K4me3 (2.7 ± 0.5 fold) histone marks, with the levels of H3K4me1 and H3K36me3 remaining relatively stable throughout the time course of *Kras*^*G12D*^ induction (Fig. 1b, c). These changes were similarly observed in other iKras cell lines tested (9805, 1012 Additional file 1: Fig S1). Silent chromatin likewise increased in overall levels with H3K27me3 (2.9 ± 0.3 fold, *p* value < 0.01) and H3K9me3 (1.7 ± 0.1 fold, *p* value < 0.05; Fig. 1b, c and Additional file 1: Fig S1). We hypothesized that beyond the global changes of histone marks measured by western blot, there is a redistribution of histone marks in the genome that leads to epigenetic alterations with the ability to be targeted and antagonize the *Kras*^*G12D*^ effect, a hypothesis that we explored using multi-omics methodologies.

**Fig. 2:**
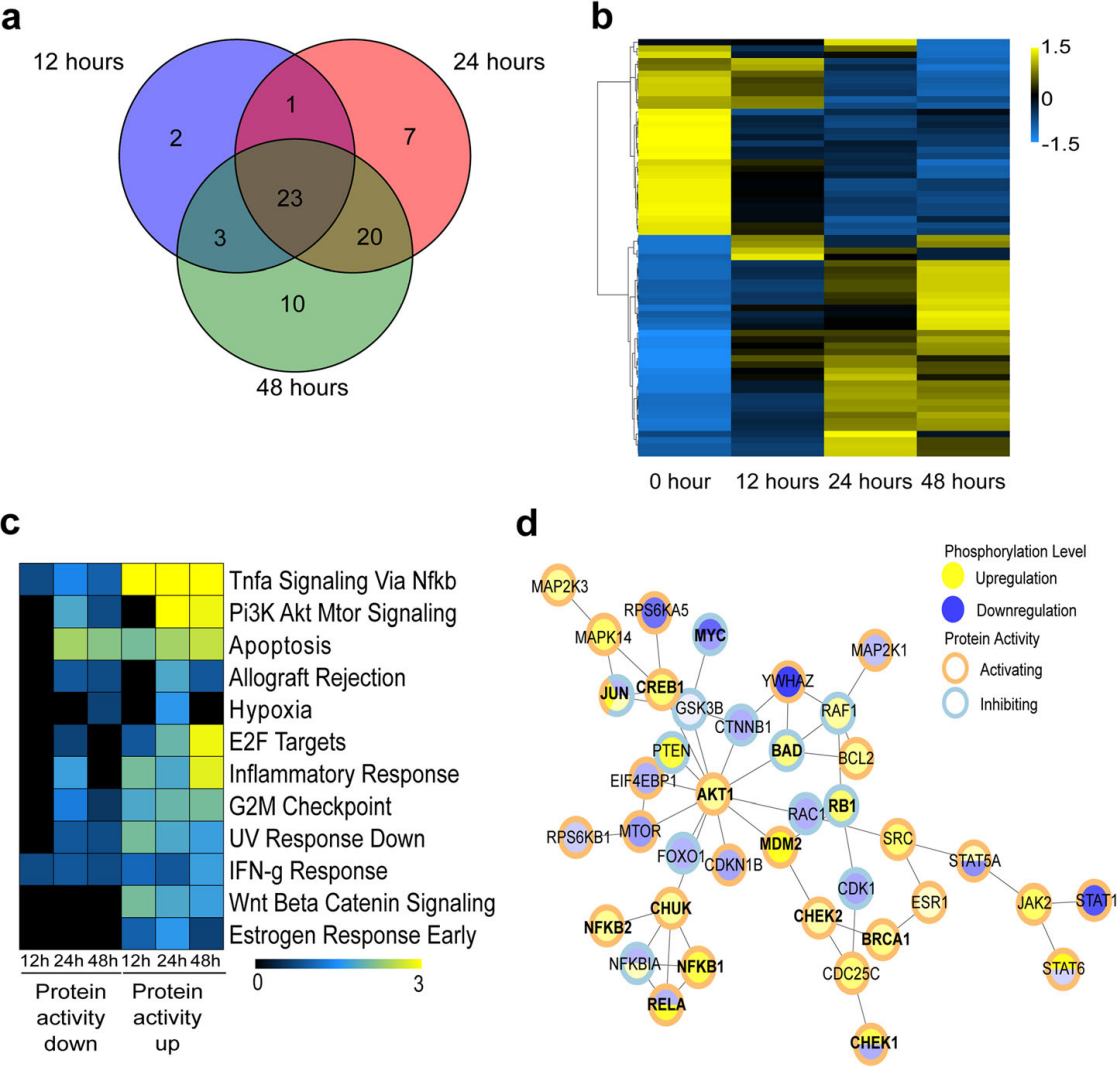
Analysis of cancer signaling phospho-antibody array containing 269 antibodies following oncogenic Kras^G12D^ induction. **a** Venn diagram of differentially phosphorylated proteins (DPPs) at 12, 24, and 48 hrs with signal fold change ≥ |1.5|. **b** Signal ratios of DPPs normalized to the Z scale and plotted for 0, 12, 24, and 48 hrs. **c** Pathway enrichment analysis of DPPs for 12, 24, and 48 hrs using the Molecular Signatures Database (MSigDB) hallmark gene set collection. Color scale represents standardized –log10(FDR) values. **d** Protein-Protein interaction network obtained from DPP using the STRING database and interaction confidence score ≥ 999. Center blue circle: downregulated protein phosphorylation log_2_(fold change). Center yellow circle: upregulated protein phosphorylation log_2_(fold change). Some proteins have multiple phosphorylation sites and, hence, will have multiple colors in the center circle. Outer concentric circles represent activating (orange) or inhibiting (light blue) protein activity. The effect on activity by the combination of the mark’s effect and its level were interpreted. For instance, an inhibitory mark decreasing should lead to a more active protein. Some proteins have both inhibitory and activating phosphorylations and, hence, will have multiple colors in the outer concentric circle. Protein names highlighted in bold are mentioned in the text with their fold changes

Next, to assess signaling phosphorylation events induced by *Kras*^*G12D*^, we used a proteomic approach through phase-absorbed phospho-antibody (PAPA) arrays. Analysis of phospho-protein densitometric signals at 0, 12, 24, and 48 hrs after *Kras*^*G12D*^ induction revealed a total of 66 differential protein phosphorylations (Fig. 2a, b). *Kras*^*G12D*^ induction by western blot (Fig. 1c) shows moderate expression and levels continuing to increase out to 24 hrs. By mirroring these time points in the PAPA array, we may miss early signaling events, but we can observe phosphorylation changes that are initiated at 12 hrs and maintained by *Kras*^*G12D*^ in these cells. There were 23 phosphorylation states in common for all three time points, whereas 2, 7, and 10 were exclusive to 12, 24, and 48 hrs, respectively (Fig. 2a). Comparing the signal ratios of protein phosphorylation across the time points demonstrates a complete reversal of the phosphorylation states between 0 and 48 hrs, with intermediate values following this trend at 12 and 24 hrs (Fig. 2b). We then categorized the protein phosphorylation status using two criteria, for each time point, based on their functional effect: (1) whether the magnitude of phosphorylation increased or decreased and (2) whether they are known to be activating or inhibiting modifications. Phosphorylation states were categorized as “activity down” if either an activating protein phosphorylation decreased in magnitude, or an inhibitory phosphorylation increased (Fig. 2c, d, Additional file 2: Table S1). Phosphorylation states were categorized as “activity up” if an activating phosphorylation increased in magnitude, or an inhibitory phosphorylation decreased. We performed pathway enrichment analysis of these two groups for each time point using the R package Rapid Integration of Term Annotation and Network resources (RITAN) [5] and the Molecular Signatures Database (MSigDB) hallmark gene set collection [6]. We confirmed that in these cell models, Kras^G12D^ induced processes related to cell growth and survival via the PI3K-AKT-mTOR-NF-κB pathway (Fig. 2c, d). We also identified induction of signals for cell proliferation that include those which are G2/M checkpoint proteins and E2F targets (Fig. 2c, d), marking the arrival of the Kras^G12D^ signal to the nucleus. Congruently, 24 hrs after *Kras*^*G12D*^ induction, we noted activation of early response gene products, such as ELK-1 (pSer383, fold change = 1.6) and JUN (pSer73, fold change = 2.46), as well as activation of immediate early genes like MYC by downregulation of inhibitory phosphorylation (pSer62, fold change = 0.4, pThr58, fold change = 0.6) (Fig. 2d, bold letters and Additional file 2: Table S1) [7–11]. Additionally, downstream of MYC, phosphorylation of RB1 (pSer780, fold change = 1.8) (Fig. 2d, bold letters and Additional file 2: Table S1) releases E2F to mediate cell cycle progression and DNA synthesis [12, 13]. Another important pathway, recapitulated by our cell model, was that of PI3K/AKT signaling, including PDK1 (pSer241, fold change = 1.5) [14]. Cells also indicated an activated AKT1 (pSer473, fold change = 1.7; pThr208, fold change = 1.5) to promote cell survival by inhibitory phosphorylation events of BAD (pSer112, fold change = 1.6; pSer115 fold change, = 1.7) [15, 16] (Fig. 2d, bold letters and Additional file 2: Table S1). Next, evidence of AKT1 activation of MDM2 (pSer166, fold change = 2.6) was seen, which inhibits p53 [17] (Fig. 2d and Additional file 2: Table S1) as well as triggers pro-survival signals by NF-κB, through IKK-α (pThr23, fold change = 1.6) [18–20]. Interestingly, we found activation of several subunits of NF-κB (RELA, pThr254, fold change = 2.4; NFKB1, pSer893, fold change = 2.2; NFKB2, pSer869, fold change = 1.6) (Fig. 2d, bold letters and Additional file 2: Table S1). Lastly, we detected markers for oncogene-mediated replication stress, such as CHEK1 (pSer317, fold change = 2.1), CHEK2 (pThr68, fold change = 1.5), and BRCA1 (pSer1423, fold change = 1.5; pSer1524, fold change = 1.7) [21] (Fig. 2d, bold letters and Additional file 2: Table S1). As a control, western blots confirm the upregulation of phosphorylated AKT1, CDC25C, and NF-κB and downregulation of inhibitory MYC phosphorylation in the 4292, 9805, and 1012 *Kras*^*G12D*^ cell lines at 48 hrs (Additional file 1: Fig S2). Thus, this data validates the usefulness of our cell models by showing that *Kras*^*G12D*^ induces a proliferative phenotype, by triggering the canonical cytoplasmic signaling that reaches the nuclei to likely regulate, in a comprehensive and coordinated manner, a chromatin-mediated effect, which we investigate below.

### *Kras*^*G12D*^ changes histone chromatin accessibility and induces mobilization of histone marks to coding and non-coding areas of the genome

**Fig. 3:**
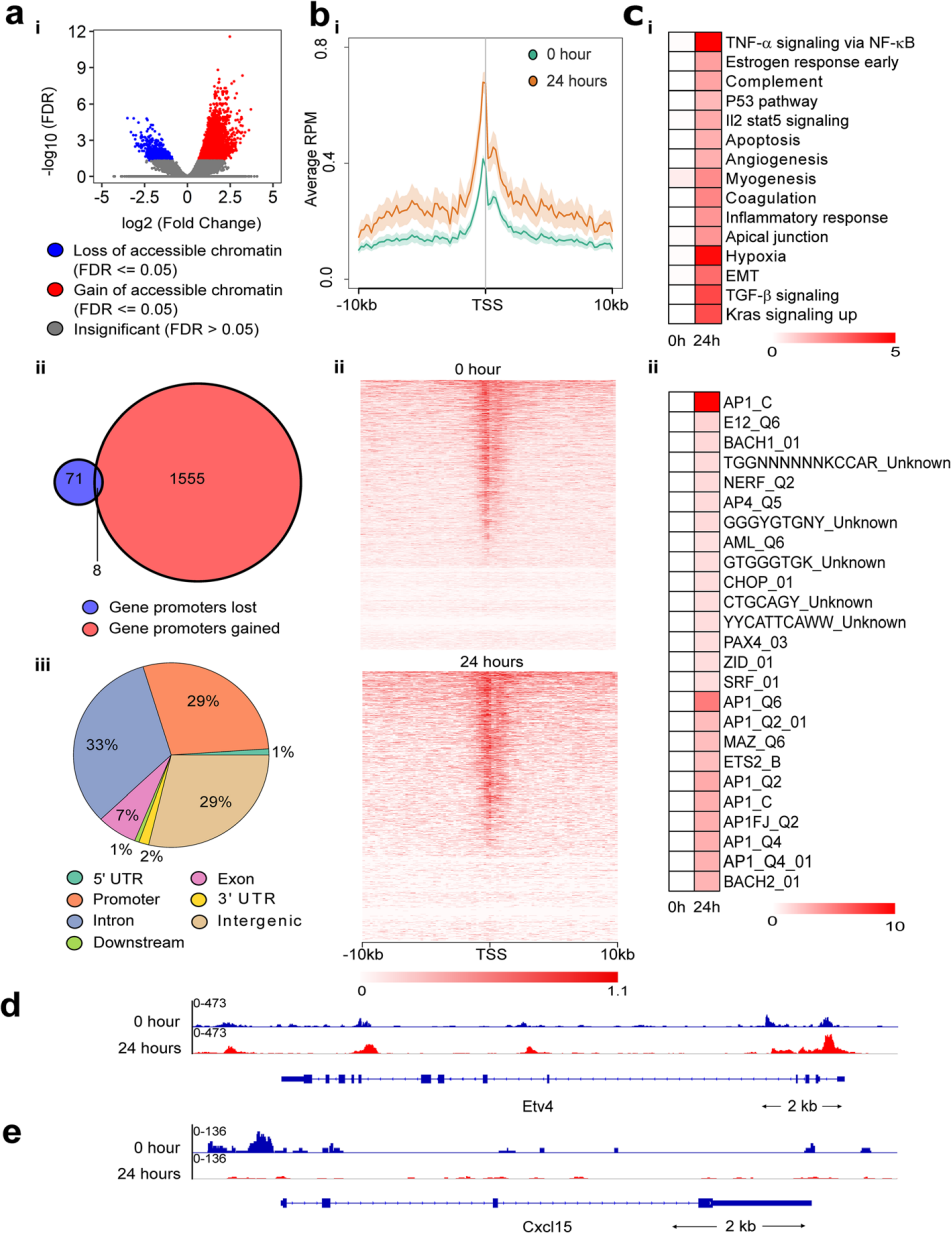
Global changes in accessible chromatin following oncogenic *Kras*^*G12D*^ induction. **a** (i) ATAC-seq was used to measure chromatin accessibilities. Each point represents a region of accessible chromatin comparing *Kras*^*G12D*^ off (0 hr) versus on (24 hrs). **a** (ii) Venn diagrams comparing the number of gene promoters gained or lost in differentially accessible regions at 24 hrs. **a** (iii) Pie graphs showing the genomic distribution of the significantly differentially accessible regions. **b** (i) Average profile plot of normalized reads ± 10 kb around gene transcription start sites (TSSs). *Y*-axis represents read count per million (RPM) mapped reads. Orange and green shaded areas represent the standard error of the mean. **b** (ii) Heatmap of normalized reads around the TSS for each gene for 0 and 24 hrs. **c** (i) Pathway enrichment and (ii) transcription factor enrichment analysis of genes annotated to accessible chromatin for 0 and 24 hrs. Color scale represents standardized −log10(FDR) values. The tags following the transcription factors in **c** (ii) represent the TRANSFAC database nomenclature, which indicates either a different motif and/or the quality of the data that was used to make the motif. **d**, **e** Normalized ATAC-seq read coverage tracks for: **d** *Etv4* (upregulated RNA-seq gene) and **e** *Cxcl15* (downregulated RNA-seq gene)

Using ATAC-Seq, we identified areas of chromatin that are remodeled in *Kras*^*G12D*^*-*induced cells. These analyses revealed 6123 regions that gain and 559 regions that lost accessibility (FDR ≤ 0.05; Fig. 3a, i). Most regions that change accessibility were in introns (33%), promoters (within 10 kb, 29%), and intergenic (29%) (Fig. 3a, iii). Hence, a characteristic of the *Kras*^*G12D*^ response is predominantly a gain in accessible chromatin, affecting 2711 genes (2564 in their introns, 1563 in promoters, from 3595 to 1830 peaks, respectively), while only 323 genes became inaccessible (312 in their introns, 79 in promoters, from 346 and 82 peaks, respectively; Fig. 3a, ii). In fact, when considering the ± 10-kb region surrounding gene transcription start sites (TSS), the global chromatin accessibility in *Kras*^*G12D*^-induced cells increased the most in areas adjacent to the TSS (7.7% increase in the −1 kb promoter region and 10.4% in first intron) (Fig. 3b, i–ii). Thus, we investigated whether the genes within newly opened chromatin begin to account for the oncogenic signal induced by *Kras*^*G12D*^. Genes mapped to NF-κB and growth regulatory expression networks (Fig. 3c, i), encoding transcription factors that work downstream of these pathways, including AP1, ETS2, SRF, and MAZ [22–24] (Fig. 3c, ii). Figure 3d–e shows two examples of TSS accessibility changing after *Kras*^*G12D*^ induction at single gene loci; the upregulated *Etv4* gene has increased accessibility and the *Cxcl15* locus shows decreased accessibility.

**Fig. 4:**
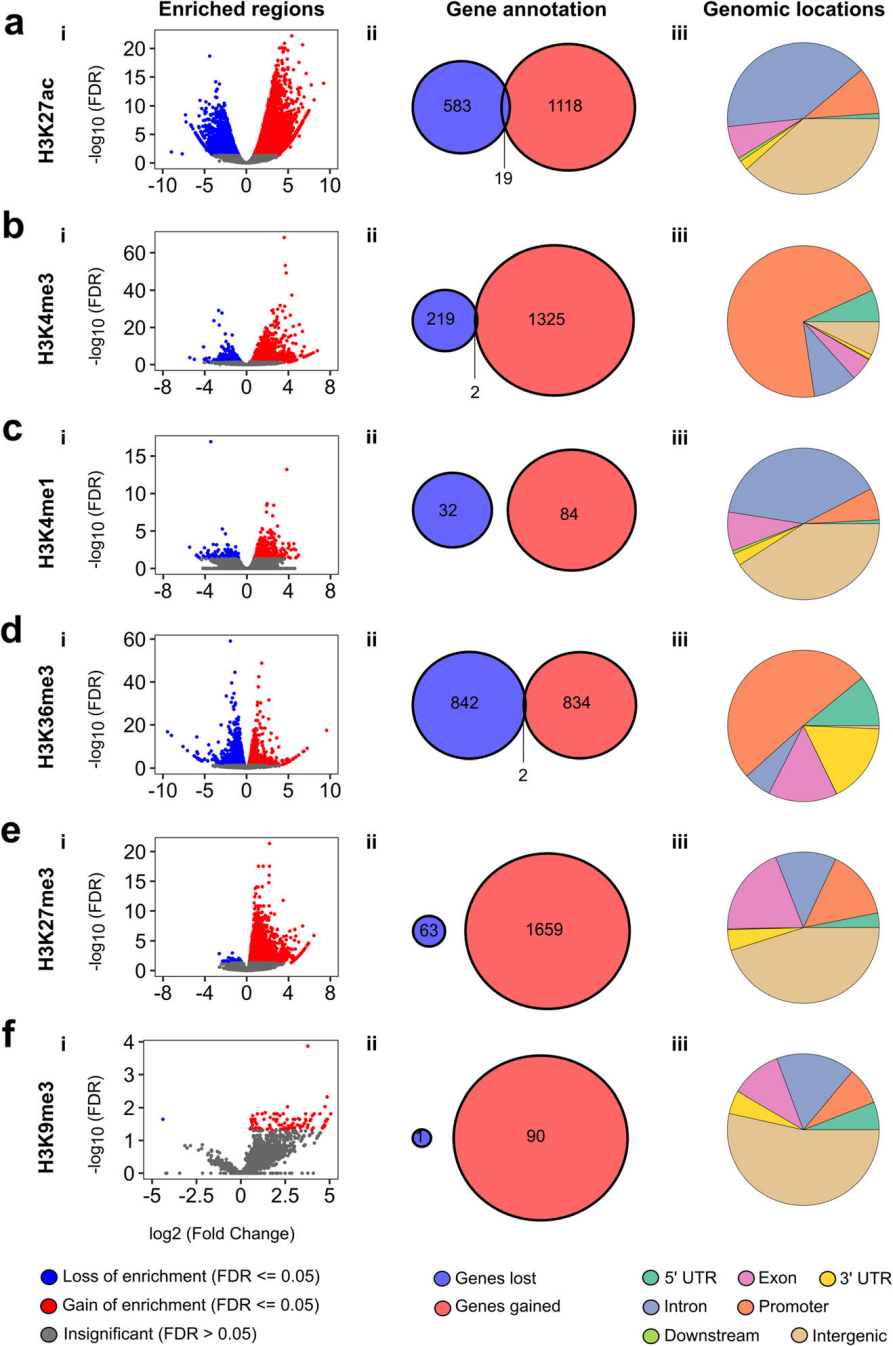
Global changes in histone marks following oncogenic *Kras*^*G12D*^ induction. **a** H3K27ac, **b** H3K4me3, **c** H3K4me1, **d** H3K36me3, **e** H3K27me3, **f** H3K9me3. **a** (i)–**f** (i): Each point represents a binding region comparing *Kras*^*G12D*^ off (0 hr) versus on (24 hrs) condition. Loss or gain of enrichment set by an FDR ≤ 0.05. **a** (ii)–**f** (ii) Venn diagrams comparing the number of genes gained or lost in differentially bound regions at 24 hrs. **a** (iii)–**f** (iii) Pie graphs showing the genomic distribution of the significantly differentially bound regions

**Fig. 5:**
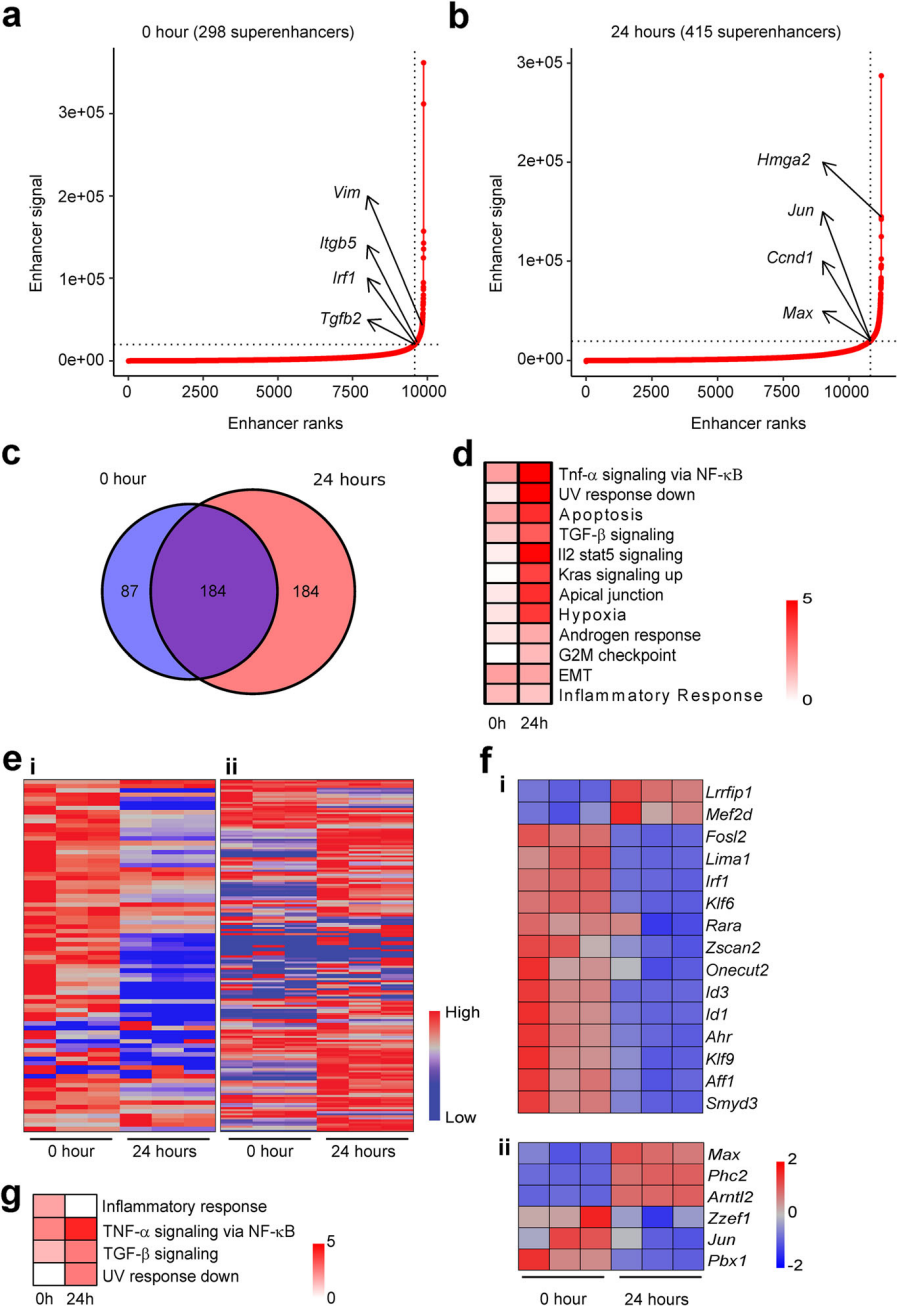
Identification of super-enhancers following *Kras*^*G12D*^ induction. **a, b** Super-enhancers identified at **a** 0 hr and **b** 24 hrs. **c** Venn diagram of super-enhancers annotated with genes comparing unique and shared super-enhancers at 0 and 24 hrs. **d** Pathway enrichment analysis of annotated super-enhancers for 0 and 24 hrs using the MSigDB hallmark gene set collection. Color scale −log10 (FDR). **e** Gene expression heatmap of all genes associated with super-enhancers at (i) only 0 hr (87 genes) and (ii) only 24 hrs (184 genes). **f** Gene expression heatmap of the transcription factors associated with super-enhancers at (i) only 0 hr and (ii) only 24 hrs. RPKM values were normalized to the *Z* scale. **g** Pathway enrichment analysis of transcription factors for 0 and 24 hrs using MSigDB hallmark gene set collection. Color scale −log10 (FDR)

Due to the fact that these open areas of chromatin may correspond to different epigenomic regulatory areas, such as promoters, enhancers, and super-enhancers, we next performed ChIP-seq on the six histone marks for both *Kras*^*G12D*^ negative (0 hr) and *Kras*^*G12D*^*-*positive (24 hrs) cells (Fig. 1b, c). ChIP-Seq for H3K27ac revealed an overall gain in enrichment of differentially marked regions, which mapped to specific genes and were located at intronic, intergenic and promoter regions after 24 hrs of *Kras*^*G12D*^ expression in cells (Fig. 4a, i–iii). We found a similar enrichment in differentially marked regions around gene TSSs for H3K4me3, which in our data set marked most promoters responsive to *Kras*^*G12D*^ (Fig. 4b, i–iii). In contrast, *Kras*^*G12D*^ had little effect on mobilizing the H3K4me1 mark, which was differentially bound at fewer gene loci as compared to previous marks and was present at intergenic, intronic, and promoter regions (Fig. 4c, i–iii). In response to *Kras*^*G12D*^, the H3K36me3 mark became equally divided between areas that show gain or loss of enrichment mapped to promoters, 3′UTRs, exons, and 5′UTRs (Fig. 4d, i–iii). Next, we studied marks that associate with inactive chromatin. We found that the facultative heterochromatin mark, H3K27me3, locates primarily outside of promoters in intergenic regions upon *Kras*^*G12D*^ expression (Fig. 4e, iii), which is congruent with published cell biology data that show accumulation of non-promoter regions and heterochromatin at the nuclear periphery and lamina-associated domains (LADs) [25–27]. The other heterochromatic mark, H3K9me3, demonstrated a modest enrichment at differentially marked regions with *Kras*^*G12D*^ at 24 hrs (Fig. 4f, i), mapping to 90 genes enriched and to 1 gene depleted (Fig. 4f, ii). These sites mostly mapped to intergenic, intronic, and exonic regions of the genome (Fig. 4f, iii). Promoters were poorly represented within H3K9me3 rich areas, consistent with a role for this mark in heterochromatic regions to form centromeres and membrane-to-heterochromatin attachment regions [26, 28]. *Kras*^*G12D*^ activation also increased super-enhancers from 298 at 0 hr (Fig. 5a) to 415 after 24 hrs (Fig. 5b). When super-enhancers were mapped to genes, 87 were unique at 0 hr versus 184 at 24 hrs, with an additional 184 overlapping, although depending on the time point, signal intensity varied (Fig. 5c and Additional file 2: Table S2). We found that unique super-enhancers, as predicted using the ROSE algorithm [29, 30], before *Kras*^*G12D*^ expression (0 hr) (Fig. 5d) were near genes regulating the mesenchymal phenotype and interferon responses, while super-enhancers after *Kras*^*G12D*^ expression (24 hrs) were near genes encoding transcription factors that mediate oncogenic functions related to proliferation and cell cycle progression [31–38] (Fig. 5d). Super-enhancer formation also encompassed regulating pathways downstream of *Kras*^*G12D*^, including RAS, NF-κB, and G2M checkpoint-mediated gene expression networks [6, 39] (Fig. 5d). An independent ChIP assay performed on all three *Kras*^*G12D*^ cell lines (4292, 9805 and 1012) in the promoter regions of *Btc, Etv4, Cdkn1a,* and *Npm1* showed an increase in the deposition of H3K27ac and H3K4me3 marks and a decrease in the deposition of those same marks at *Itgb5, Pdgfrb, Wnt10b,* and *Uba7*. This was confirmation that *Kras*^*G12D*^ induction leads to the same remodeling of activating chromatin marks as seen with ChIP-seq analysis (Additional file 1: Fig S3). These results indicate that super-enhancers make an important contribution to the Kras^G12D^ response and its phenotypic transitions.

We next investigated how these *Kras*^*G12D*^ induced super-enhancer changes related to gene expression changes, proximally and via transcription factors. We plotted expression levels (reads per kilobase per million mapped, RPKM) of the 87 genes proximal to super-enhancers that are present only at 0 hr (Fig. 5e, i) and the 184 genes associated to those present only at 24 hrs (Fig. 5e, ii). Generally, genes associated with super-enhancers have higher expression compared to the same genes when the super-enhancers were not formed. This consistent change in gene expression supports a clear functional role for these changes in super-enhancer architecture, rapidly after oncogene induction. Next, we investigated how many of the super-enhancer-associated genes exclusive to each time point were transcription factors and had significant changes in gene expression, to better understand how super-enhancer assembly and disassembly could signal throughout the genome. At 0 hr (no *Kras*^*G12D*^), super-enhancers associated with 15 different transcription factors, out of which 13 were highly expressed and then downregulated by *Kras*^*G12D*^ induction at 24 hrs (Fig. 5f, i). Notably, these transcription factors are all regulators of cell differentiation and xenobiotic metabolism [40–44]. There were 6 transcription factors associated with super-enhancers at only 24 hrs in *Kras*^*G12D*^ and half of them had corresponding transcriptional upregulation at 24 hrs (Fig. 5f, ii). Therefore, gene expression was relatively concordant with super-enhancer regulation and further implicated transcription factors could be initiating genome-wide reorganization after super-enhancer remodeling. Ongoing studies that consider the impact of three-dimensional chromatin structure and regulation of super-enhancers on more distal genes will elucidate additional levels of regulation that may explain differences from the simple model that super-enhancers always upregulate cis-acting genes. Transcription factors among the differentially expressed genes at both time points function in various signaling pathways necessary for immune and oncogenic functions (Fig. 5g). Therefore, *Kras*^*G12D*^ expression triggers an active remodeling of super-enhancers, which act in concert with the expression of transcription factors to contribute to the effects of this oncogene. Furthermore, combined ATAC-Seq and ChIP-Seq data demonstrates that Kras^G12D^ induces a robust global remodeling of primarily activating chromatin marks for enhancers (H3K27ac), promoters (H3K4me3), and gene bodies (H36Kme3), with less of a contribution from H3K4me1. On the other hand, heterochromatin reorganization (H3K27me3, H3K9me3) is less associated with promoters, suggesting that these marks play an additional role apart from a direct influence on transcription, as revealed below by integrative analyses of all our omics datasets. These changes are a dominant feature associated to the *Kras*^*G12D*^ phenotype in this GEMM-derived cell model.

### *Kras*^*G12D*^ induces rapid and global changes in DNA methylation, primarily at promoter regions and distinct repetitive areas of the genome

**Fig. 6:**
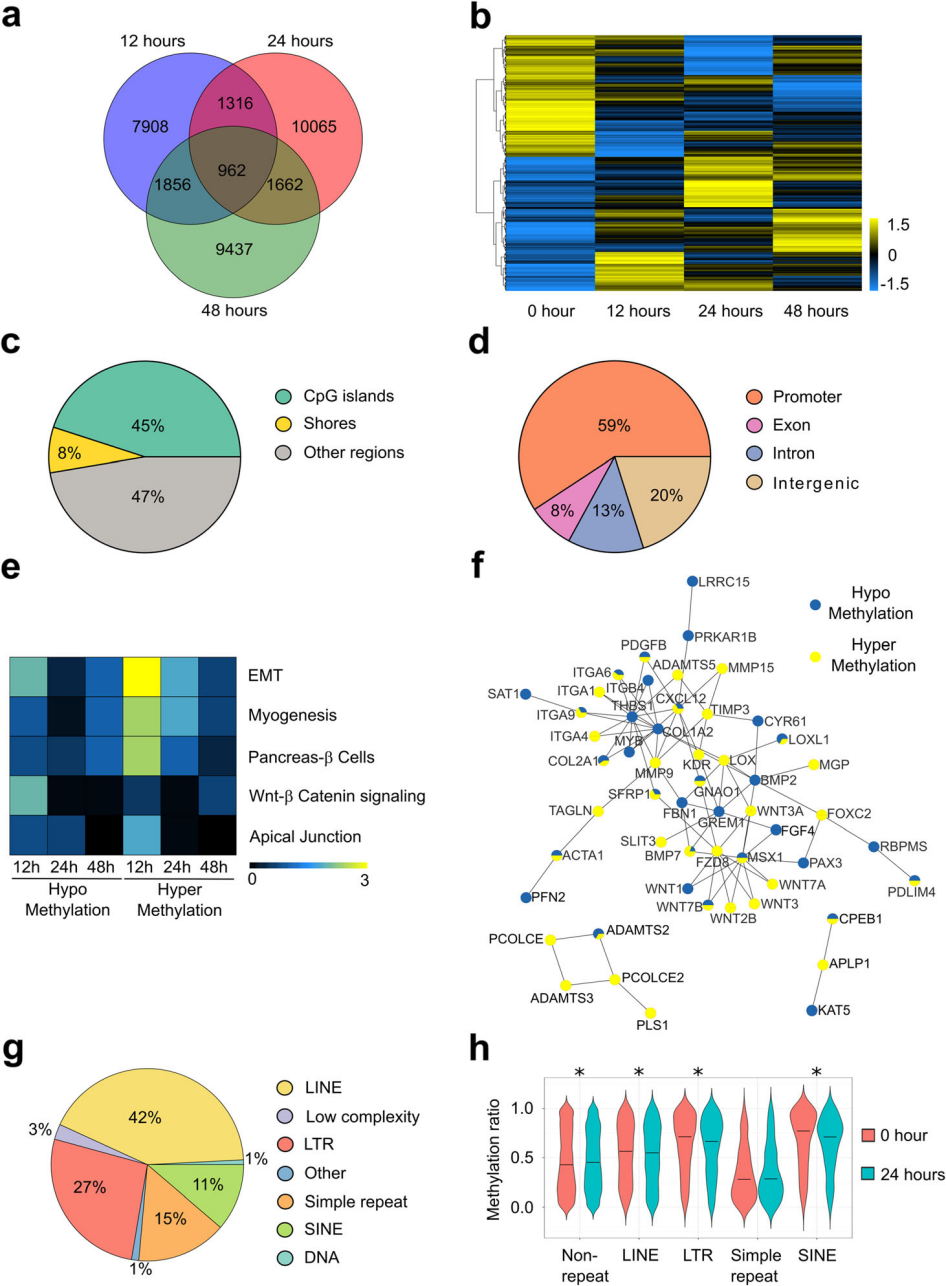
Identification of DNA methylation changes following oncogenic *Kras*^*G12D*^ induction using RRBS. **a** Venn diagram of unique and overlapping differentially methylated cytosines (DMCs) for 12, 24, and 48 hrs. DMCs were classified based on *p* value < 0.01 and methylation difference > |10%|. **b** Methylation ratio of DMCs normalized to the *Z* scale and plotted for 0, 12, 24, and 48 hrs. Yellow: hypermethylated DMC. Blue: hypomethylated DMC. Black: no change. **c** Annotation of DMCs with CpG islands and shores. **d** Annotation of DMCs with genic features. **e** Pathway enrichment analysis of DMCs located within ± 3 kb of gene transcription start sites (TSSs) for 12, 24, and 48 hrs using the MSigDB hallmark gene set collection. Color scale: −log10(FDR). **f** Protein-protein interaction network obtained from DMCs for the epithelial to mesenchymal transition (EMT) pathway using the STRING database. Confidence score: 700. **g** Distribution of DMCs within various repetitive element types. **h** Methylation ratio of DMCs distributed within non-repeats and repetitive elements. Non-repeat *n* = 10,864, LINE *n* = 1411, LTR *n* = 791, Simple repeat *n* = 465, SINE *n* = 347. Wilcoxon signed rank test was used to test for differences between 0 and 24 hrs for each category. * significant at *p* value < 0.05

**Fig. 7:**
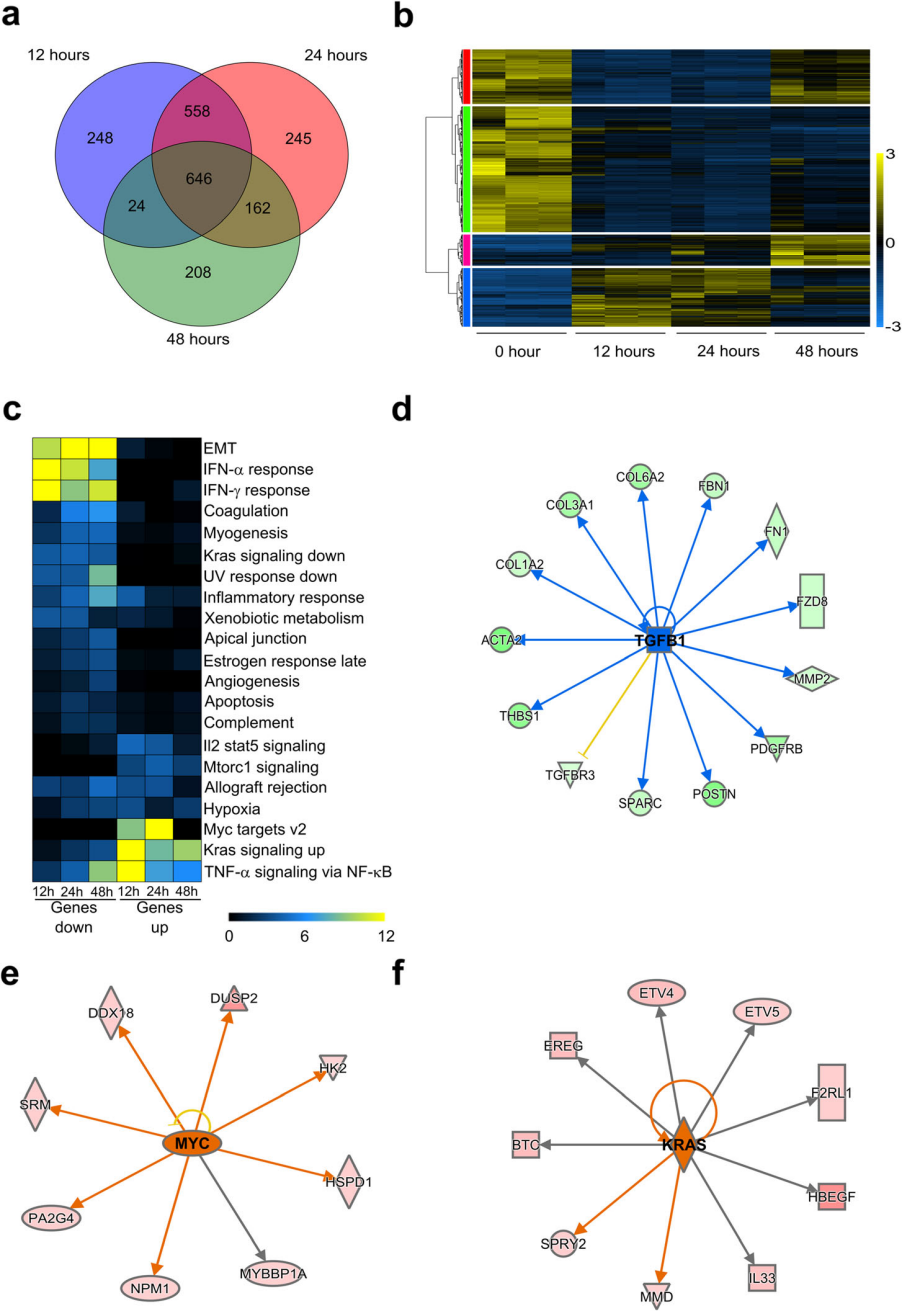
RNA-seq analysis following oncogenic *Kras*^*G12D*^ induction. **a** Venn diagram of differentially expressed genes (DEGs) at 12, 24, and 48 hrs. DEGs were classified based on expression fold change ≥ |2| and FDR ≤ 0.05. Total number of DEGs across time points: 2091. **b** RPKM expression levels of DEGs normalized to the *Z* scale and plotted for 0, 12, 24, and 48 hrs. Yellow: positive change. Blue: negative change. Black: no change. **c** Pathway enrichment analysis of DEGs for 12, 24, and 48 hrs using RITAN and MSigDB hallmark gene set collection. Color scale − log10(FDR). Gene expression networks obtained from upstream regulatory analysis at 24 hrs in Ingenuity Pathway Analysis (IPA) software for downregulated **d** TGFB1, and upregulated **e** MYC and **f** KRAS. The blue and green gene shapes and lines indicate predicted or observed inhibition respectively, while the yellow line indicates the predicted relationship is inconsistent with gene expression. The orange and red gene shapes and lines indicate predicted activation or observed upregulation respectively. Gray lines indicate no predicted effect

Due to the fact that many of the histone-based pathways work in coordination with 5-cytosine methylation of CpGs, we performed reduced representation bisulfite sequencing (RRBS) [45] (Additional file 2: Table S3). We found that *Kras*^*G12D*^ deploys a rapid and robust methylation response with thousands of differentially methylated CpGs (DMCs) identified in *Kras*^*G12D*^-expressing cells (Fig. 6a, b). More than half of these DMCs were in CpG islands or flanking shores (Fig. 6c). Further annotation revealed that majority of these modifications occurred within promoters, followed by intergenic, intronic, and exonic regions (Fig. 6d). Pathway enrichment analysis of genes annotated to DMCs upon *Kras*^*G12D*^ induction revealed that overall hypermethylation with concomitant gene silencing of promoters were primary involved in epithelial-to-mesenchymal transition (EMT), congruent with transcriptional silencing of genes in this pathway identified by RNA-seq experiments (Fig. 6e, f genes with yellow circles, Fig. 7c) [46–49].

We next analyzed methylation of repetitive elements such as long interspersed nuclear elements (LINE), short interspersed nuclear element (SINE), and long terminal repeats (LTR), which can be differentially methylated in cancers and linked to genomic instability [50]. The majority of the DMCs found in *Kras*^*G12D*^*-*expressing cells were in non-repetitive elements, although a significant number of LINE, LTR, simple repeats, and SINE were also represented (Fig. 6g and Additional file 1: Fig S4a). Changes in DMCs within non-repeats and repetitive elements revealed that LINE are hypermethylated in *Kras*^*G12D*^ cells at 12 hrs (Additional file 1: Fig S4b), but hypomethylated, along with LTR and SINE elements, after 24 hrs (Fig. 6h). At 48 hrs, LINE and SINE elements continued to be hypomethylated, whereas hypermethylation marked simple repeats (Additional file 1: Fig S4c). DMCs in non-repetitive regions became hypermethylated in *Kras*^*G12D*^ cells at 24 and 48 hrs (Fig. 6h and Additional file 1: Fig S4c). Thus, we conclude that *Kras*^*G12D*^ causes an overall decrease in methylation of LINE, SINE, and LTR by elements*,* which has yet to be recognized in response to this oncogene. Combined, these analyses demonstrate that *Kras*^*G12D*^ triggers a rapid methylation response that varies in genomic location, although quantitatively similar through time. Simultaneously, *Kras*^*G12D*^ causes demethylation of repetitive elements that associate to pathways such as reactivation of endogenous retroelements and genomic instability [51]. Thus, these phenomena are an important reflection of the potential of Kras^G12D^ to participate in different aspects of carcinogenesis, such as genomic instability, which may underlie the accumulation of genetic alterations in pancreatic tissues over time, increasing the likelihood for a second hit.

### Transcriptional outcome of the combined remodeling of nuclear functions induced by *Kras*^*G12D*^

RNA-seq, performed at 0, 12, 24, and 48 hrs after doxycycline treatment, detected 2091 differentially expressed genes (DEG) (Fig. 7a and Additional file 2: Table S4). A total of 646 DEGs were identified at all post-induction time points. However, distinct subsets only changed at specific times, with 248, 245, and 208 DEGs at 12, 24, and 48 hrs, respectively (Fig. 7a). Hierarchical clustering defined four expression patterns (Fig. 7b, color bars). Two patterns (labeled red and green) were primarily represented by transcripts that were downregulated in *Kras*^*G12D*^ cells at 12 hrs and remained low through 24 hrs (1395 genes). At 48 hrs, 421 genes in the red pattern were upregulated, separating from the green pattern. The third pattern (labeled pink) contained 241 genes minimally expressed at 0 hr, but gradually increased to their maximum expression at 48 hrs. Finally, the 455 genes in the fourth pattern (labeled blue) increased in expression at 12 hrs and returned to near-basal levels by 48 hrs. Thus, in contrast with changes to chromatin, where we found a gain of enrichment for marks involved in transcriptional activation, nearly two-thirds of the DEGs exhibited robust downregulation. Pathway enrichment analyses using RITAN indicated that, at all three time points, downregulated genes, which included transcripts such as *Snai2*, *Acta2*, and multiple collagen genes, participate in EMT (Fig. 7c) [47]. Another set of downregulated genes were represented by the hallmark interferon alpha and gamma responses (Fig. 7c). Other algorithms such as IPA and cluster Profiler show that these pathways ranked among the top 5 in *Kras*^*G12D*^ cells at 12 and 24 hrs (Additional file 1: Fig S5 and S6). Genes exclusively upregulated by *Kras*^*G12D*^ at 12 and 24 hrs were enriched for proliferation and survival pathways, such as Myc targets and Mtorc1 signaling, both involved in protein synthesis and growth [52–57] (Fig. 7c and Additional file 1: Fig S5 and S6). Using upstream regulatory analysis (URA), we found *Tgfb1* as a key node for regulating a mesenchymal network (Fig. 7d and Additional file 1: Fig S7). URA also shows that *Kras*^*G12D*^ induction maintained the proliferative and growth-related *MYC* and *KRAS* gene networks at 24 hrs (Fig. 7e, f and Additional file 1: Fig S7). *Kras*^*G12D*^ induction increased expression of EGF-like pathways [58], which reinforce its oncogenic potential [59–61] (Additional file 2: Table S4). To confirm the gene expression patterns and consistency of alterations in key pathways, transcriptomics were completed on the 1012 and 9805 cell lines (Additional file 2: Table S4). Filtered to match the set of 2091 genes altered by the 4292 cells, we observed that approximately 85% of the time the direction of the DEG was matched across all 3 cell lines (Additional file 1: Fig S8). Additionally, genes of the IFN-alpha, IFN-gamma, EMT, Myc, Mtorc1, and Kras up-/downregulation were similarly altered in these three *Kras*^*G12D*^-inducible cell lines. In summary, RNA-seq complements our cellular studies at a quantitative level by showing that *Kras*^*G12D*^ induces the downregulation of a sizable percentage of gene networks with selective upregulation of those which promote oncogenicity.

### Integrative analyses uncover single and combinatorial chromatin events underlying the early transcriptional response to *Kras*^*G12D*^

**Fig. 8:**
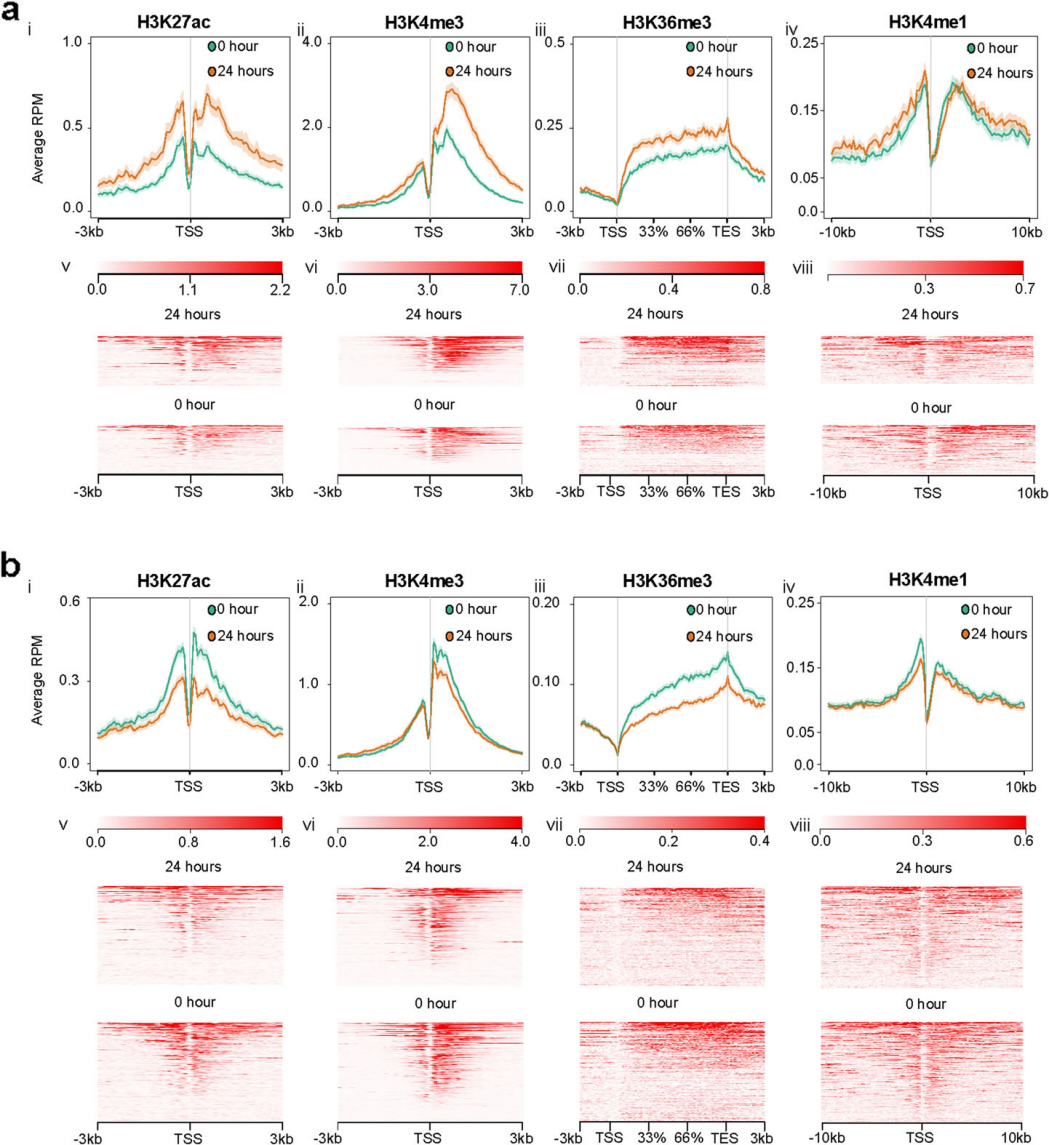
Chromatin marks at gene transcription start sites and gene bodies correlate with up- and downregulated transcripts following *Kras*^*G12D*^ induction. **a** (i–iv) Average profile plots of normalized H3K27Ac, H3K4Me3, H3K36Me3, and H3K4me1 reads around the transcription start site (TSS) and gene body for upregulated genes at 24 hrs in the RNA-seq data (446 genes). Orange and green shaded areas represent the standard error of the mean. **a** (v–viii) Red heatmaps show normalized reads around the TSS or gene body for each upregulated gene. **b** (i–iv) Average profile plots of normalized H3K27Ac, H3K4Me3, H3K36Me3, and H3K4me1 reads around gene TSS and the gene body for downregulated genes at 24 hrs in the RNA-seq data (1165 genes). Color scheme is same as **a** (i–iv). **b** (v–viii) Red heatmaps show normalized reads around the TSS or gene body for each downregulated gene

**Fig. 9:**
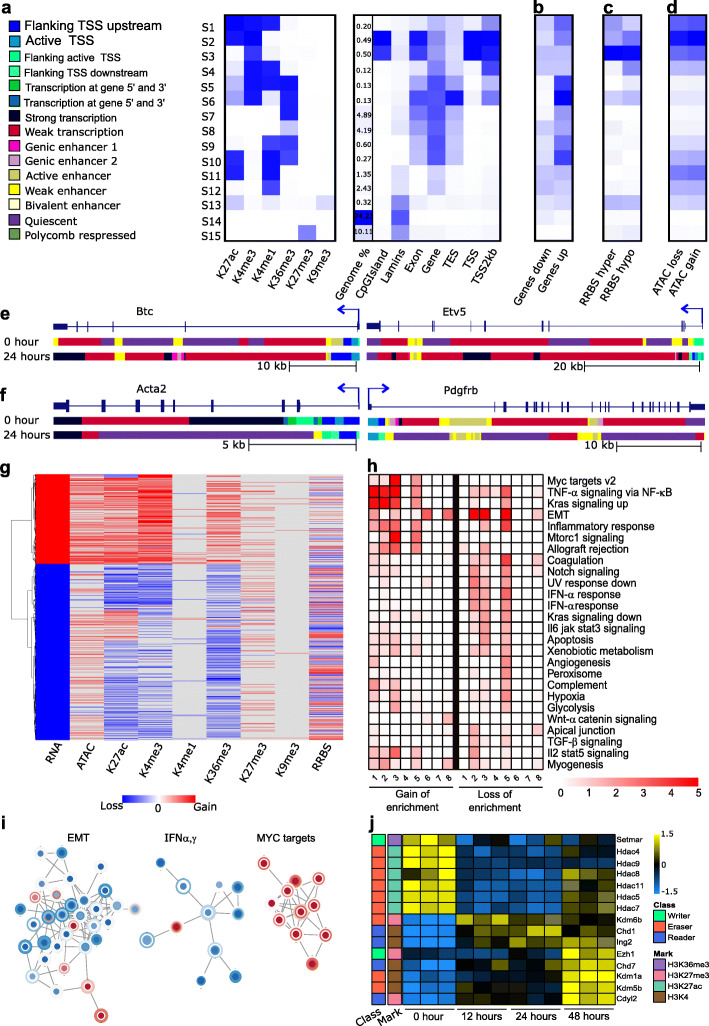
Multi-omics data integration reveals single and combinatorial chromatin remodeling events that affect gene transcription and cell phenotype. **a** ChromHMM segmentation of the genome. TES: Transcription end site, S: States 1–15. Integration of ChromHMM with **b** RNA-seq, **c** DNA methylation, and **d** ATAC-seq. Chromatin segmentation of **e** two upregulated genes, *Btc* and *Etv5* and **f** two downregulated genes, *Acta2* and *Pdgfrb* illustrating the change in marks at 0 and 24 hrs. **g** Heatmap displaying log2 fold changes of genes in the RNA-seq, ATAC-seq, ChIP-seq, and methylation ratio of RRBS data set at 24 hrs. **h** Pathway enrichment analysis of genes represented in panel **g** using RITAN. Gain or loss of enrichment: RNA-seq genes enriched in (1) ATAC-seq (2), H3K27ac (3), H3K4me3 (4), H3K4me1 (5), H3K36me3 (6), H3K27me3 (7), H3K9me3, and (8) hypermethylated (gain), hypomethylated (loss) gene promoters respectively. **i** Gene networks generated from the genes represented in **g**. Center red or blue dots represent upregulated and downregulated genes in the RNA-seq data set respectively. Concentric circles around the dots represent changes in H3K27ac, H3K4me3, and H3K36me3 respectively (going inside out) with red representing gain of the mark and blue representing loss of the mark.  Enlarged figure with gene details provided as Additional file 1: Fig S10. **j** RPKM expression levels of differentially expressed histone readers, writers, and erasers normalized to the *Z* scale and plotted for 0, 12, 24, and 48 hrs

Our integrative analyses were initiated by filtering RNA-seq data and overlaying this data with peaks derived from ChIP-seq. We plotted H3K27ac, H4K4me3, H3K36me3, and H3K4me1 ChIP-seq signals for DEGs obtained from our RNA-seq data set in *Kras*^*G12D*^ cells at 24 hrs (Fig. 8). We found an increase in H3K27ac and H3K4me3 at gene TSSs and H3K36me3 within gene bodies (Fig. 8a, i–iii). In fact, two-thirds of the genes expressed in *Kras*^*G12D*^ cells displayed enrichment of these 3 marks at 24 hrs compared to 0 hr (Fig. 8a, v–vii). Concomitantly, nearly two-thirds of genes downregulated at 24 hrs in *Kras*^*G12D*^*-*expressing cells exhibited a decrease in these marks (Fig. 8b, i–iii, b, v–vii). In contrast, changes observed in other marks, such as H3K4me1 (Fig. 8a, iv, viii, b, iv, viii), H3K27me3, and H3K9me3 (Additional file 1: Fig S9), were not significantly enriched at promoter or enhancer regions and thus likely do not participate in transcriptional initiation. Moreover, we integrated information from ChIP-seq, ATAC-seq, and RRBS, using ChromHMM, to develop a model with 15 distinct chromatin states (States 1–15) as outputs, annotated as previously reported [62–64]. These analyses confirmed that active TSSs or flanking TSSs were marked by combinations of H3K4me3 with H3K27ac and H3K4me1 (Fig. 9a; States 1–4). Surprisingly, these regions combined only represent 1.3% of the entire genome. Of similar importance, these marks were absent in 74% of the genome (Fig. 9a; State 14). Thus, this data provides insights into the existence of an early *Kras*^*G12D*^-associated epigenomic landscape that accounts for the transcriptional outcome, which at a global and quantitative level facilitate the remodeling of euchromatic vs. repressive chromatin.

This pattern of histone-based chromatin remodeling correlated with expression of upregulated genes in RNA-seq, hypomethylation of DNA promoters by RRBS, and increased accessible chromatin regions by ATAC-seq (Fig. 9b–d; State 2). States 5 and 6, which represent transcription of genes at 5′ and 3′ ends, were marked with H3K4me3, H3K27ac, H3K4me1, and H3K36me3, constituting 0.26% of the genome (Fig. 9a; States 5–6). Nine percent of the genome was also enriched in H3K36me3 reflecting the ability of *Kras*^*G12D*^ to induce an active program of post-initiation, transcriptional processing within gene bodies (Fig. 9a, b; States 7–8). Enhancer regions were the primary remodeling target of *Kras*^*G12D*^ and comprised about 5% of the genome marked by both H3K4me1 and H3K27ac (Fig. 9a; States 9-13). Another important finding is that 10% of the genome was marked by H3K9me3 or H3K27me3 (Fig. 9a; States 13 and 15 respectively), which as suggested by ChromHMM, relocated to LADs (Fig. 9a; States 13–15) in response to *Kras*^*G12D*^ induction. Mapping the ChromHMM states along a 2D linear gene representation depicts how chromatin states alternate along segments of DNA, although they likely represent contacts that these genes make with chromatin proteins which aggregate in distinct 3D domains [65]. For instance, two upregulated genes, *Btc* and *Etv5*, were part of the proliferative network observed in RNA-seq (Fig. 7f), which gained transcriptionally active chromatin and lost the quiescent state within the gene body in *Kras*^*G12D*^ cells (Fig. 9e). Under the same conditions, *Etv5* gained marks of an active TSS. Conversely, close examination of genes downregulated at 0 hr, representing networks regulated by *Tgfb1* (*Acta2* and *Pdgfrb*) (Fig. 7d), demonstrated that their gene bodies lost marks and acquired a quiescent chromatin state upon *Kras*^*G12D*^ induction (Fig. 9f). We also integrated RNA-seq expression patterns with all epigenomic data sets by a hierarchical clustering-based method which illustrated that *Kras*^*G12D*^ cells displayed a total of 859 DEGs with upregulated genes mapping to accessible chromatin regions (ATAC-seq) and enrichment for H3K27ac, H3K4me3, and H3K36me3 marks (Fig. 9g). Conversely, downregulated genes primarily located at accessible chromatin regions lost enrichment of H3K27ac, H3K4me3, and H3K36me3. However, both up- and downregulated genes demonstrated an overall increase in H3K27me3. Pathway enrichment analyses on the integrated epigenomics dataset found that genes within accessible ATAC-seq regions and triple-marked by H3K27ac, H3K4me3, and H3K36me3 participate in cell survival, growth, and proliferative pathways (Fig. 9h). Conversely, genes that lost H3K27ac, H3K4me3, and H3K36me3 but gained H3K27me3 and underwent promoter hypermethylation primarily belong to pathways that can aid cells to acquire a pro-oncogenic capacitation by *Kras*^*G12D*^ (Fig. 9h). We also built interacting gene expression networks of loci modified by different epigenomic pathways, overlaying fold changes in RNA expression (center circle) with the changes in H3K27ac, H3K4me3, and H3K36me3 (Fig. 9i and Additional file 1: Fig S10). Most downregulated genes that control EMT as well as IFN-α and IFN-γ responses exhibited depletion of at least one of the activating histone marks (Fig. 9i and Additional file 1: S10). Conversely, upregulated genes that control ribosomal biogenesis through Myc were enriched in at least one of these marks (Fig. 9i and Additional file 1: Fig S10). Interestingly, analyses of the effects of *Kras*^*G12D*^ on key epigenomic regulators that are known to impact the marks studied here demonstrated the downregulation of histone deacetylases (HDACs), enzymes which remove the H3K27ac mark, in order to dissemble enhancers and super-enhancers (Fig. 9j). Hence, these changes are congruent with a hierarchical and critical role of enhancers and super-enhancers and, potentially, some regulators (e.g., HDAC, histone acetyltransferases (HATs)) to initiate transcription in response to *Kras*^*G12D*^. In summary, this study integrates knowledge derived from multiple datasets, generated in a controlled and time-dependent manner, to account for many features shown to characterize the *Kras*^*G12D*^ phenotype in GEMM-derived pancreatic cells.

## Discussion

The current study extends our understanding of how the epigenome functions as an effector of early *Kras*^*G12D*^ signaling. Using an extensive battery of state-of-the-art multi-omics methodologies, we have used a step-wise design to follow the impact of this oncogene on levels of chromatin marks and their genome localization for 6 different histone marks, as well as its impact on chromatin accessibility, DNA methylation, and the transcriptome. The analyses of results from each of these datasets were mapped initially to the entire genome (global remodeling), without taking into consideration their impact on transcription. Subsequently, the integration of all the data together provided information on how distinct changes in the epigenome associate or not with the transcriptional response to *Kras*^*G12D*^. The new knowledge derived from these experiments represents a robust characterization of these oncogene-driven events in a pancreatic cell model. Consequently, it is important to summarize and discuss these findings in light of the role that this oncogene has in preparing pancreatic cells to begin the transition toward abnormal cell growth.

The choice of cell model for the study was carefully considered since previous studies have demonstrated that pancreatic tumor cells are heterogenous [1]. Thus, we first validated the usefulness of GEMM-derived pancreatic cells carrying an inducible *Kras*^*G12D*^ allele as an appropriate model for our study. These modifications were accompanied by changes in a phenotypic transition from a slow proliferative cell to a more epithelioid proliferative one. Using PAPA arrays, we defined that the extensive signaling cascade triggered by *Kras*^*G12D*^ was congruent with its biological effect and complemented the analyses that support the use of these GEMM-derived cells as a model for studying the early epigenomic landscape of Kras^G12D^.

While several studies seeking to understand the role of *Kras*^*G12D*^-mediated effects in pancreatic cells using multi-omic approaches have emerged and provide useful data, our current investigation has the advantage of describing a controlled, time-dependent, coordinated, and comprehensive design, following the signal from the oncogene to the moment it enters into the nucleus to remodel the epigenome. This allows us to build a “landscape” for the early epigenomic response to *Kras*^*G12D*^, which should be a key resource for future mechanistic studies to determine the role of a myriad of writers, readers, and erasers of both DNA methylation and the histone code. First, we analyzed the data in a general manner, seeking to understand the total remodeling of the epigenome, independent of considering the impact on transcription. Subsequently, we mapped the key events that can account for the early oncogenic transcriptional response of *Kras*^*G12D*^. These results are important, since most work in this area has been directed to underscore transcriptionally linked chromatin-remodeling events. However, epigenomic changes without an apparent link to direct transcriptional control may still influence gene expression through changes in nuclear structure and dynamics. Further support for this idea is given by the fact that the transcriptional response to *Kras*^*G12D*^ is dominated by gene silencing events, including for transcription factors which likely have trans-activity, while ATAC-Seq shows the largest effects on chromatin opening. However, heterochromatin marks (H3K9Me3 and H3K27Me3) do not appear as major direct contributors to the transcriptional outcome. Instead, we suggest, and is predicted by ChromHMM and published cell biology data, that heterochromatin marks primarily undergo relocation to the nuclear periphery and LADs [25–27], rather than with mRNA synthesis and processing. Therefore, *Kras*^*G12D*^ mounts a transcriptional response primarily characterized by gene expression silencing, for which the underlying mechanism cannot be attributed to reduced chromatin accessibility, DNA methylation, nor heterochromatin formation on transcriptionally active genes.

Notably, we find that most of the transcriptional output in response to *Kras*^*G12D*^ signaling can be accounted for by a hierarchical cascade of changes that begin with the formation of enhancers and super-enhancers to influence the function of gene promoters and bodies. Hence, our study both confirms and extends a previous report performed in GEMM using an elegant approach, which concluded that the metastatic potential of PDAC cells correlates with changes in enhancers reflected by a global H3K27ac enrichment [2]. Interestingly, although human and mice PDAC might differ in many aspects, work from our laboratory has also demonstrated a key role for enhancers and super-enhancers in enabling the acquisition of specific pancreatic cancer subtypes [64]. Hence, these studies expand our understanding of chromatin remodeling in response to *Kras*^*G12D*^ signaling and may help to delineate potential evolutionarily conserved mechanisms for oncogenesis from mice-to-human. Interestingly, examination of the expression of writers, readers, and erasers of both DNA and histone-based pathways demonstrate that these changes primarily correlate with the downregulation of HDACs. This observation is relevant since these molecules can deacetylate the H3K27ac mark for enhancers and super-enhancers, which are necessary to trigger and maintain other epigenomic events.

We should also consider potential impact of the current study for future biomedical experiments in mice, as well as the search for novel therapeutic strategies. Indeed, many of the writers, readers, and erasers of both DNA methylation and the histone code are emerging as important mechanistic nodes in cancer development as well as promising new therapeutic targets [66]. Emerging in vivo studies using GEMM are finding that some nuclear regulators known to either deposit, bind, or reverse both DNA and/or histone marks impact KRAS^G12D^ initiation [2]. However, the field is just at an early stage and a deeper, exhaustive understanding of the role of the epigenome as an effector of oncogenes will remain a matter of intensive investigations [65].

## Conclusion

In conclusion, outlining how all the pathways studied here work in concert and in a time-regulated manner to mediate the effects of the most common initiating mutation for PDAC is important for helping to interpret mechanistic experiments and inspire the exploration of future therapeutic directions. We demonstrate that *Kras*^*G12D*^ has a quantitatively larger effect on transcriptional activation via remodeling euchromatin-associated histone pathways while its role in repression is rather passive, by decreasing their level and reorganization. We also describe, however, chromatin remodeling events which, although not directly associated with transcriptional output, are part of the Kras^G12D^ epigenomic landscape likely necessary for achieving its full oncogenic potential. *Kras*^*G12D*^-induced chromatin remodeling in GEMM pancreatic cells, as captured using multi-omics, shows the early nuclear response and describes how changes in gene expression are brought about by this oncogene, bearing both mechanistic and potential medical relevance due to promising and emerging experimental therapeutics targeting these pathways. Thus, this new knowledge should be taken into consideration when planning future mechanistic studies directed toward antagonizing early effects of *Kras*^*G12D*^, a critical step for inhibiting cancer initiation.

## Experimental procedures

### Tissue culture and reagents

iKras cell lines 4292, 9805, and 1012 were maintained in complete media (RPMI, Gibco 11875 with 10% fetal bovine serum and 1 μg/mL doxycycline) to continually express constitutively active Kras^G12D^ as described [4, 67]. Cell lines were provided by Dr. Marina Pasca di Magliano and have regularly tested negative for mycoplasma with the last test occurring in July 2020. For RNA, DNA, ATAC, and ChIP experiments, cells were grown for 48–72 hrs without doxycycline to inactivate expression of Kras^G12D^. To initiate the time course, cells were counted and split to 5E5 cells per 100 mm dish and allowed to attach overnight. The following morning, 0 hr (no *Kras*^*G12D*^ expression) was collected per RNA/DNA/ChIP protocols and doxycycline (1 μg/mL) was added to media and allowed to incubate for 12, 24, and 48 hrs prior to harvest. The inducible *Kras*^*G12D*^ expression system utilized here does not allow for the elimination of any confounding effects that doxycycline treatment alone may initiate on the transcriptome and epigenome of these cells. For proliferation assays, cells were plated at 1000 cells per well in a 96-well plate, doxycycline added to experimental groups and then incubated in the IncuCyte S3 with images captured every 6 hrs. Cell confluence was calculated using the IncuCyte software, with each condition normalized to 0 hr and fold changes calculated between doxycycline (*Kras*^*G12D*^ expressing) and no doxycycline condition at each time point.

### Western blot

iKras proteins were collected from cells at all time points after *Kras*^*G12D*^ induction by lysis in RIPA buffer (20 mM Tris-HCl (pH 7.5) 150 mM NaCl, 1 mM Na_2_EDTA, 1 mM EGTA, 1% NP-40, 1% sodium deoxycholate), supplemented with phosphatase and protease inhibitors (Thermo Fisher Scientific). Equal amounts of protein were electrophoresed on 12% SDS-PAGE gels and transferred to nitrocellulose membranes (GE Healthcare). Membranes were blocked in either 5% milk or 3% BSA for 1 hr, and primary antibody incubations were performed overnight at 4 °C with rocking. Additional file 2: Table S5 contains primary antibodies and dilutions used. Anti-mouse or anti-rabbit secondary antibodies (1:5000, Millipore) were incubated on the membranes for 1 hr at room temperature with shaking, followed by detection of bands with chemiluminescence (Thermo Fisher Scientific). Uncropped versions of all western blots are available (Additional file 1: Fig S11, Fig S12, Fig S13). Quantification of bands in three independent biological experiments was completed using ImageJ and statistical significance determined by Student’s *t* tests in GraphPad Prism.

### Phospho-antibody array

Cancer Signaling Phospho-Antibody Array from Full Moon BioSystems were used per manufacturer recommendations and sent back to the company for scanning and quantification. The protein phosphorylation state ratio was defined as the signal intensity of phospho site-specific antibody/signal intensity of site specify antibody. The ratio change between samples was defined as the treatment sample/control sample. RITAN [5] and the Molecular Signatures Database (MSigDB) hallmark gene set collection [6] were used to perform pathway enrichments.

### Assay for transposase-accessible chromatin sequencing (ATAC-seq)

iKras 4292 pellets of 5E3 cells were collected at 0 and 24 hrs and resuspended in ATAC buffer and processed as described by Volk et al. [68]. Briefly, the genome was fragmented by tagmentation, DNA fragments purified, and sequencing and indexing primers added by PCR. Additional cycles of amplification, ranging from 10 to 14 cycles, was completed to minimally increase library content. Libraries were recovered with the MinElute PCR Cleanup kit (Qiagen, 28004) and size selected for fragment sizes of 100–500 bp with AMPure XP beads (Beckman Coulter, A63881). Sequencing was completed by the GSPMC at the Medical College of Wisconsin on the Illumina HiSeq-2500 with 126 bp paired end read length and an average of 125 million paired end reads per sample, completed in biological triplicate. The ATAC-seq pipeline from Encode consortium was used for adapter trimming, read alignment, and peak calling of libraries [69]. Briefly, paired end reads were mapped by Bowtie using the mm10 reference genome. ATAC-seq peaks were called using MACS2 software at *p* value ≤ 0.01. The R package DiffBind was used to identify differentially accessible regions with FDR ≤ 0.05. As defined in DiffBind, the coordinates and the size of these regions were defined by obtaining the narrowest region of overlapping peaks. The identified regions were annotated with genes if they were located within ± 10 kb of gene transcription start sites (TSSs) using the R package ChIPseeker [70]. R package RITAN and the Molecular Signatures Databases [6, 71] was used to perform pathway and transcription factor enrichment analysis. To identify global differences in accessible chromatin at each time point, ngsplot software was used to generate normalized tag density profiles around ± 10 kb of gene transcription start sites. ATAC-seq reads around specific genes were visualized using the integrative genomics viewer (IGV) [72]. Prior to visualization, reads were normalized (RPKM) using the deepTools software [73].

### ChIP-seq

At time points of 0 and 24 hrs, DNA-protein interactions were crosslinked in 4292 iKras cells using 10% formaldehyde. The reaction was quenched with glycine and cells harvested in PBS containing protease inhibitors. Chromatin was sheared, immunoprecipitated, and processed as previously described in Lomberk et al. [64]. Supernatant was incubated with various histone mark antibodies (Additional file 2: Table S6) and protein G-agarose beads (Roche 11719416001) for 3 days with mixing at 4 °C. Following DNA purification, samples underwent real-time PCR at positive and negative loci and confirmed histone mark enrichment over 1% input. Sequencing was completed at the Mayo Clinic Medical Genomics Core on the Illumina HiSeq 2000 with 51 bp paired end and 25 million reads per sample, completed in biological duplicate. Data was analyzed using the HiChIP pipeline [74]. Briefly, paired end reads were mapped by BWA [75] using the mm10 reference genome and pairs with one or both ends uniquely mapped were retained. H3K4me3, H3K4me1, and H3K27ac peaks were called using the MACS2 software package [76] at FDR ≤ 1%. SICER [35] was used to identify enriched domains for H3K36me3, H3K27me3, and H3K9me3. The R package DiffBind [77, 78] was used to identify differentially bound regions (DBRs) with FDR ≤ 0.05. The DBRs were annotated with genes using the R package ChIPseeker [70]. The following cut-offs from the transcription start site was used for gene annotation: H3K27ac (± 10 kb), H3K4me3 (± 3 kb), H3K36me3 (− 3 kb,10 kb), H3K4me1 (± 10 kb), H3K27me3 (any distance from TSS), H3K9me3 (any distance from TSS). The ROSE software [29, 30] was used to identify stitched enhancers and to separate super-enhancers from typical enhancers based on K27ac bam files and peak files. The stitching distance used was 12,500 kb. Regions around TSS 2500 kb were not considered while calling super-enhancers. R package RITAN [79] and the MSigDB hallmark gene set collection [6] was used to perform pathway enrichment analysis of genes annotated to super-enhancers.

### qPCR of histone marks at specific ChIP locations

At time points of 0 and 24 hrs, DNA-protein interactions were crosslinked in 4292, 9805, and 1012 iKras cells in triplicate and prepared for ChIP assay as described above. The immunoprecipitation was performed against H3K4me3 and H3K27ac marks (Additional file 2: Table S6). BED files generated by H3K4me3 and H3K27ac ChIP-seq performed on 4292 iKras were imported to IGV and enriched bound regions were selected for primer design (Additional file 2: Table S7). As sample reference, we utilized 1% of input for all ChIP performed, and to normalize the samples, we employed the following standard equation: 1% input = 2 ^ [(mean Ct input − 6.64) − mean Ct ChIP] × 100. The results are shown as fold change (sample/control).

### Reduced representation bisulfite sequencing (RRBS)

At each time point (0, 12, 24, and 48 hrs), cells were washed once in PBS, detached with a scraper in 1 mL PBS, pelleted, and preserved at −20 °C. DNA was isolated from cell pellets using the QIAamp DNA Mini and Blood Mini Kit (Qiagen, 51304). A single biological condition was collected and provided to the Mayo Clinic Medical Genomics Core for bisulfite conversion, library preparation, and sequencing. Briefly, DNA (250 ng) was digested with Msp1 (New England Biolabs (NEB), R0106M) and purified using Qiaquick Nucleotide Removal Kit (Qiagen, 28004). End-repair A tailing was performed (NEB, M0212L) and TruSeq methylated indexed adaptors (Illumina, 15025064) were ligated with T4 DNA ligase (NEB, M0202L). Size selection was performed with Agencourt AMPure XP beads (Beckman Coulter, A63882). Bisulfite conversion was performed using EZ-DNA Methylation Kit (Zymo Research, D5001) as recommended by the manufacturer with the exception that incubation was performed using 55 cycles of 95 °C for 30 s and 50 °C for 15 min. Following bisulfite treatment, the DNA was purified as directed and amplified using Pfu Turbo C Hotstart DNA Polymerase (Agilent Technologies, 600414). Library quantification was performed using Qubit dsDNA HS Assay Kit (Life Technologies, Q32854) and the Bioanalyzer DNA 1000 Kit (Agilent Technologies, 5067-1504). The final libraries from RRBS were prepared for sequencing as per the manufacturer’s instructions in the Illumina cBot and HiSeq Paired end cluster kit v3. Samples were sequenced at 51 bp paired end reads using Illumina HiSeq 2000 with TruSeq SBS sequencing kit v3. Data was collected using HiSeq data collection v1.5.15.1 software, and bases were called using Illumina’s RTA v1.13.48. Raw data was further analyzed using SAAP-RRBS [80], a streamlined analysis and annotation pipeline for RRBS. Briefly, FASTQ files were trimmed to remove adaptor sequences and reads less than 15 bp were discarded. Trimmed FASTQ files were then aligned against the reference genome mm10 using BSMAP [81]. Methylation was reported along with custom CpG annotation. A minimum of five reads was required for inclusion of a cytosine in subsequent high-level analyses. Differential methylation of individual CpG loci was detected by performing a Fisher exact test with Methylkit [82] between a pair of samples at different time points (0, 12, 24, and 48 hrs) after selecting only the CpGs which have a data available across all the samples. The differentially methylated CpGs (DMCs) were selected according to the combination of *p* value (*p* value < 0.01) and absolute methylation difference > 10%. DMCs within ± 3 kb of gene TSS were used for pathway enrichment analysis using RITAN and the Molecular Signatures Database (MSigDB) hallmark gene set collection [6] for each time point. Gene networks were generated using RITAN and Cytoscape [83]. R package genomation [84] was used for DMC annotation with CpG islands and genic elements. Annotation of DMCs with repetitive elements was performed using repeat element coordinates (mm10) obtained from RepeatMasker [85] and BEDTools [86].

### RNA-seq

At each time point (0, 12, 24, and 48 hrs for 4292 and 0, 24 hrs for 1012 and 9085), cells were washed once in PBS and harvested with RLT + βME as per RNeasy Mini Kit (Qiagen, 74106). RNA isolation included an on-column DNA digestion step and yielded > 1 μg per sample. For 4292 cells, three independent biological replicates were collected and provided to the Mayo Clinic Medical Genomics Core for sequencing. RNA libraries were prepared with the Illumina TruSeq RNA v2 kit and sequencing completed on the Illumina HiSeq-2000 with 101 bp paired end reads. For 1012 and 9085 cells, two independent biological replicates were collected and provided to the Genomic Science and Precision Medicine Center (GSPMC) at the Medical College of Wisconsin. Libraries were prepared with the Illumina TruSeq RNA stranded kit and sequencing completed on the Illumina NovaSeq6000 with 100 bp pair-end reads. Raw sequencing reads were processed through the Mayo RNA-seq bioinformatics workflow, MAPR-Seq v1.2.1.3 [87]. Raw and normalized (RPKM) counts for 23,398 genes and corresponding exons, expressed single nucleotide variants (SNVs) as well as gene fusions were obtained per sample. The R package from Bioconductor, edgeR v3.8.6 [88], was used for differential analysis comparing gene expression at all time points (12, 24, and 48 hrs) to 0 hr. Prior to the analysis, lowly expressed genes (raw counts less than 30 reads) were removed. Genes with false discovery rate (FDR) less than 5% and an absolute fold change ≥ 2 were considered to be significantly differentially expressed. The Ingenuity Pathway Analysis software (IPA, Qiagen) [89], RITAN and clusterProfiler [90] software was used to analyze canonical pathways, upstream regulators, diseases, and toxicity functions using the subset of genes that were significantly differentially expressed.

### Data integration

#### RNA-ChIP integration

To identify local differences in occupancy of H3K27ac, H3K4me3, and H3K36me3 around differentially expressed genes (DEGs), we merged bam files from both replicates at 0 and 24 hrs and used ngsplot to generate normalized tag density profiles around gene TSSs or the gene body. The upregulated and downregulated DEGs were identified from 24 hrs in RNA-seq analysis.

#### Integration of ChromHMM with RNA-seq, ATAC-seq, and RRBS

We used the software ChromHMM which uses hidden Markov model to segment the genome into distinct states [91]. ChIP-sequencing replicates at each time point were merged before binarization step to learn models from the data. Fifteen states of segmentation were visualized for both 0 and 24 hrs time points. The 15-state segmentation was used to identify fold enrichment across various genomic features (note *y-*axis of fold enrichment plots such as CpG islands and refseq Exon). The coordinates for LADs were obtained from Peric-Hupkes et al. [92]. For integration of ChromHMM with RNA-seq, ATAC-seq, and RRBS, bed files containing coordinates for each region of interest were generated using the following methods: (1) RNA-seq: DEGs identified at 24 hrs were annotated with chromosome number, start and end position using the R Bioconductor package -biomaRt [93], (2) ATAC-seq: peak files for 0 and 24 hrs were sorted using BEDTools [86] and the three replicates for each time point merged using BEDOPS [94] to generate bed files, (3) RRBS: coordinates of the DMCs identified at 24 hrs (*p* value < 0.01 and methylation difference > │10%│) that were within ± 3 kb of gene TSSs were extracted into hyper- and hypomethylated bed files. ChromHMM output files containing the segmented genome for 0 and 24 hrs were used to view chromatin states of gene loci on the UCSC genome browser [95]. Heatmap of RNA-seq and combined epigenomic markers was generated using log_2_fold changes for RNA-seq, ATAC-seq, and histone marks, while methylation ratio was used for RRBS. Pathway enrichment analysis and gene networks were generated using RITAN [79], MSigDB hallmark gene set collection [6], and Cytoscape [83].

## Supplementary Information

**Additional file 1: Fig S1.** Induction of *Kras*^*G12D*^ leads to changes in global histone mark levels. **Fig S2.** Induction of *Kras*^*G12D*^ leads to changes in phosphorylation events. **Fig S3.** Induction of *Kras*^*G12D*^ leads to changes in the remodeling of activating chromatin. **Fig S4.** Annotation of DMCs with non-repeats and repetitive elements. **Fig S5.** Ingenuity Pathway Analysis (IPA) of RNA-seq genes. **Fig S6.** Gene enrichment analysis of RNA-seq genes. **Fig S7.** Upstream regulatory analysis of RNA-seq genes conducted in IPA. **Fig S8.** RNA-seq analysis following oncogenic *Kras*^*G12D*^ induction in 1012 and 9085 cell lines. **Fig S9.** Chromatin marks at gene bodies for up and downregulated transcripts following *Kras*^*G12D*^ induction. **Fig S10.** Networks generated from genes associated with H3K27ac, H3K4me3 and H3K36me3 marks, enlarged figure with gene details of Fig. 9i from the main manuscript. **Fig S11.** Uncropped version of western blots present in Fig. 1b of the main manuscript. **Fig S12.** Uncropped version of western blots present in Supplementary Figure 1. **Fig S13.** Uncropped version of western blots present in Supplementary Figure 2.

**Additional file 2: Table S1.** Signaling Phospho Antibody Array. **Table S2.** Super-enhancer Signal Intensities. **Table S3.** Statistics based on per sample analysis for RRBS. **Table S4.** RNA-seq fold changes. **Table S5.** Antibodies used for western blot. **Table S6.** Antibodies used for ChIP-seq. **Table S7.** Primers used for ChIP-seq.

**Additional file 3.** Review history.

## Data Availability

All sequencing datasets generated for this manuscript are available in the ArrayExpress public database repository [96–100] under the following accession codes: E-MTAB-10909, ATAC-seq of pancreatic cancer cells derived from a genetically engineered mouse model, which harbor the inducible Kras^G12D^ allele. E-MTAB-10901, ChIP-seq of pancreatic cancer cells derived from a genetically engineered mouse model, which harbor the inducible Kras^G12D^ allele. E-MTAB-10900, RRBS of pancreatic cancer cells derived from a genetically engineered mouse model, which harbor the inducible Kras^G12D^ allele. E-MTAB-10896, RNA-seq of pancreatic cancer cells derived from a genetically engineered mouse model, which harbor the inducible Kras^G12D^ allele. E-MTAB-10897, RNA-seq of pancreatic cancer cells (1012 and 9805) derived from a genetically engineered mouse model, which harbor the inducible Kras^G12D^ allele.

## Abbreviations

GEMMs: Genetically engineered cells and mouse models
RPM: Read count per million
TES: Transcription End Site
PAPA: phase-absorbed phospho-antibody
RE: Regulatory elements
ATAC-seq: Transposase-accessible chromatin sequencing
ChIP-seq: Chromatin immunoprecipitation followed by sequencing
RRBS: Reduced representation bisulfite sequencing
HMM: Hidden Markov modeling
LADs: Lamina-associated domains
DB: Differentially bound
TSS: Transcription start site
RPKM: Reads per kilobase million
DMCs: Differentially methylated CpGs
EMT: Epithelioid to mesenchymal transition
LINE: Long interspersed nuclear elements
SINE: Short interspersed nuclear element
LTR: Long terminal repeats
DEGs: Differentially expressed genes
RITAN: Rapid integration of term annotation and network resources
MSigDB: Molecular signatures database
IGV: Integrative genomics viewer
IPA: Ingenuity Pathway Analysis
URA: Upstream regulatory analysis
HATs: histone acetyltransferases
HDACs: Histone deacetylases
