# Supplementary material for: KrasG12D induces changes in chromatin territories that differentially impact early nuclear reprogramming in pancreatic cells
Source: Genome Biol. 2021 Oct 14;22:289. doi: 10.1186/s13059-021-02498-6 (PMC8518179; doi:10.1186/s13059-021-02498-6)
Supplement: Supplementary file 1 — Additional file 1: Fig S1. Induction of KrasG12D leads to changes in global histone mark levels. Fig S2. Induction of KrasG12D leads to changes in phosphorylation events. Fig S3. Induction of KrasG12D leads to changes in the remodeling of activating chromatin. Fig S4. Annotation of DMCs with non-repeats and repetitive elements. Fig S5. Ingenuity Pathway Analysis (IPA) of RNA-seq genes. Fig S6. Gene enrichment analysis of RNA-seq genes. Fig S7. Upstream regulatory analysis of RNA-seq genes conducted in IPA. Fig S8. RNA-seq analysis following oncogenic KrasG12D induction in 1012 and 9085 cell lines. Fig S9. Chromatin marks at gene bodies for up and downregulated transcripts following KrasG12D induction. Fig S10. Networks generated from genes associated with H3K27ac, H3K4me3 and H3K36me3 marks, enlarged figure with gene details of Fig. 9i from the main manuscript. Fig S11. Uncropped version of western blots present in Fig. 1b of the main manuscript. Fig S12. Uncropped version of western blots present in Supplementary Figure 1. Fig S13. Uncropped version of western blots present in Supplementary Figure 2. [file 13059_2021_2498_MOESM1_ESM.docx]

**Kras^G12D^ induces changes in chromatin territories that differentially impact early nuclear reprogramming in pancreatic cells**

Angela J. Mathison^1,2,9¶^, Romica Kerketta^1,2,10¶^, Thiago Milech de Assuncao^2^, Elise Leverence^1^, Atefeh Zeighami^1,3^, Guillermo Urrutia^2^, Timothy Stodola^1,2,11^, Marina Pasca di Magliano^4^, Juan L. Iovanna^5^, Michael T. Zimmermann^1,3,6,12^, Gwen Lomberk^1,2,7,13^*, and Raul Urrutia^1,2,8,14^*

^1^Genomics and Precision Medicine Center (GSPMC), Medical College of Wisconsin, Milwaukee, WI

^2^Division of Research, Department of Surgery, Medical College of Wisconsin, Milwaukee, WI Center, Medical College of Wisconsin, Milwaukee, WI

^3^Bioinformatics Research and Development Laboratory, and Precision Medicine Simulation Unit, Genomics and Precision Medicine Center (GSPMC), Medical College of Wisconsin, Milwaukee, WI

^4^Department of Surgery, University of Michigan, Ann Arbor, MI

^5^Centre de Recherche en Cancérologie de Marseille (CRCM), INSERM U1068, CNRS UMR 7258, Aix-Marseille Université and Institut Paoli-Calmettes, Parc Scientifique et Technologique de Luminy, Marseille, France

^6^Clinical and Translational Sciences Institute, Medical College of Wisconsin, Milwaukee, WI Department of Pharmacology and Toxicology, Medical College of Wisconsin, Milwaukee, WI Department of Biochemistry, Medical College of Wisconsin, Milwaukee, WI

^7^Department of Pharmacology and Toxicology, Medical College of Wisconsin, Milwaukee, WI

^8^Department of Biochemistry, Medical College of Wisconsin, Milwaukee, WI

^9^@MathisonAngie, ^10^@theysee, ^11^@TimStodola, ^12^@MTZimmermann, ^13^@GLomberk, ^14^@precision_ru

*** Corresponding authors**: Gwen Lomberk, PhD; E-mail: [glomberk@mcw.edu](mailto:glomberk@mcw.edu) and Raul Urrutia, MD; E-mail: rurrutia@mcw.edu

**Running Title**: Epigenomic Effects Downstream of *Kras^G12D^* Signaling

^¶^A.M. and R.K contributed equally to this work

**Keywords:** Pancreatic Cancer, KRAS, epigenomics

Additional File 1: **Fig S1**: Induction of *Kras^G12D^* leads to changes in global histone mark levels. **Fig S2**: Induction of *Kras^G12D^* leads to changes in phosphorylation events. **Fig S3**: Induction of *Kras^G12D^* leads to changes in the remodeling of activating chromatin. **Fig S4**: Annotation of DMCs with non-repeats and repetitive elements. **Fig S5**: Ingenuity Pathway Analysis (IPA) of RNA-seq genes. **Fig S6**: Gene enrichment analysis of RNA-seq genes. **Fig S7**: Upstream regulatory analysis of RNA-seq genes conducted in IPA. **Fig S8**: RNA-seq analysis following oncogenic *Kras^G12D^* induction in 1012 and 9085 cell lines. **Fig S9**: Chromatin marks at gene bodies for up and downregulated transcripts following *Kras^G12D^* induction. **Fig S10**: Networks generated from genes associated with H3K27ac, H3K4me3 and H3K36me3 marks. **Fig S11**: Uncropped version of western blots present in Figure 1B of the main manuscript. **Fig S12**: Uncropped version of western blots present in Supplementary Figure 1. **Fig S13**: Uncropped version of western blots present in Supplementary Figure 2.


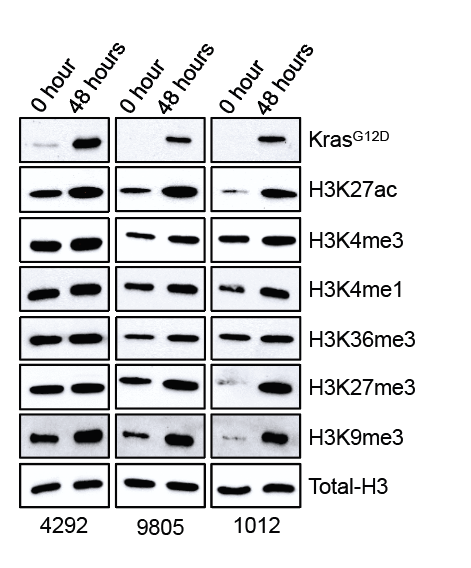


**Fig S1. Induction of *Kras^G12D^* leads to changes in global histone mark levels.** Western blot analysis was performed in all i*Kra*s cell lines (4292, 9805 and 1012) at 0 and 48 hours. Cell lysates were probed with the indicated antibodies for histone marks.


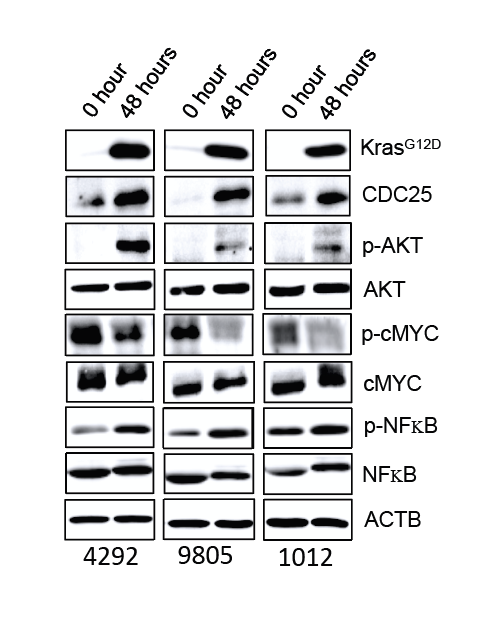


**Fig S2. Induction of *Kras^G12D^* leads to changes in phosphorylation events.** Western blot analysis was performed in all i*Kra*s cell lines (4292, 9805 and 1012) at 0 and 48 hours time point. Cell lysates were probed with the indicated antibodies confirming the results shown by PAPA.


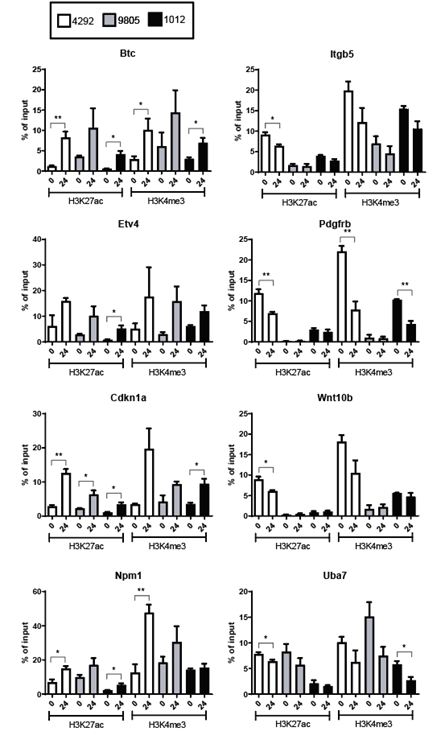


**Fig S3. Induction of *Kras^G12D^* leads to changes in the remodeling of activating chromatin.** ChIP assay was performed in all i*Kra*s cell lines (4292, 9805 and 1012) at 0 and 24 hours time point. *Btc, Etv4, Cdkn1a* and *Npm1* showed an increase in the deposition of H3K4me3 and H3K27ac mark and *Itgb5*, *Pdgfrb*, *Wnt10b* and *Uba7* showed a decrease in the deposition of H3K4me3 and H3K27ac mark upon Kras^G12D^ induction. * indicates p-value ≤0.05, and ** indicates p-value ≤0.005. All data is expressed as mean ±SEM (experiment performed in triplicate).


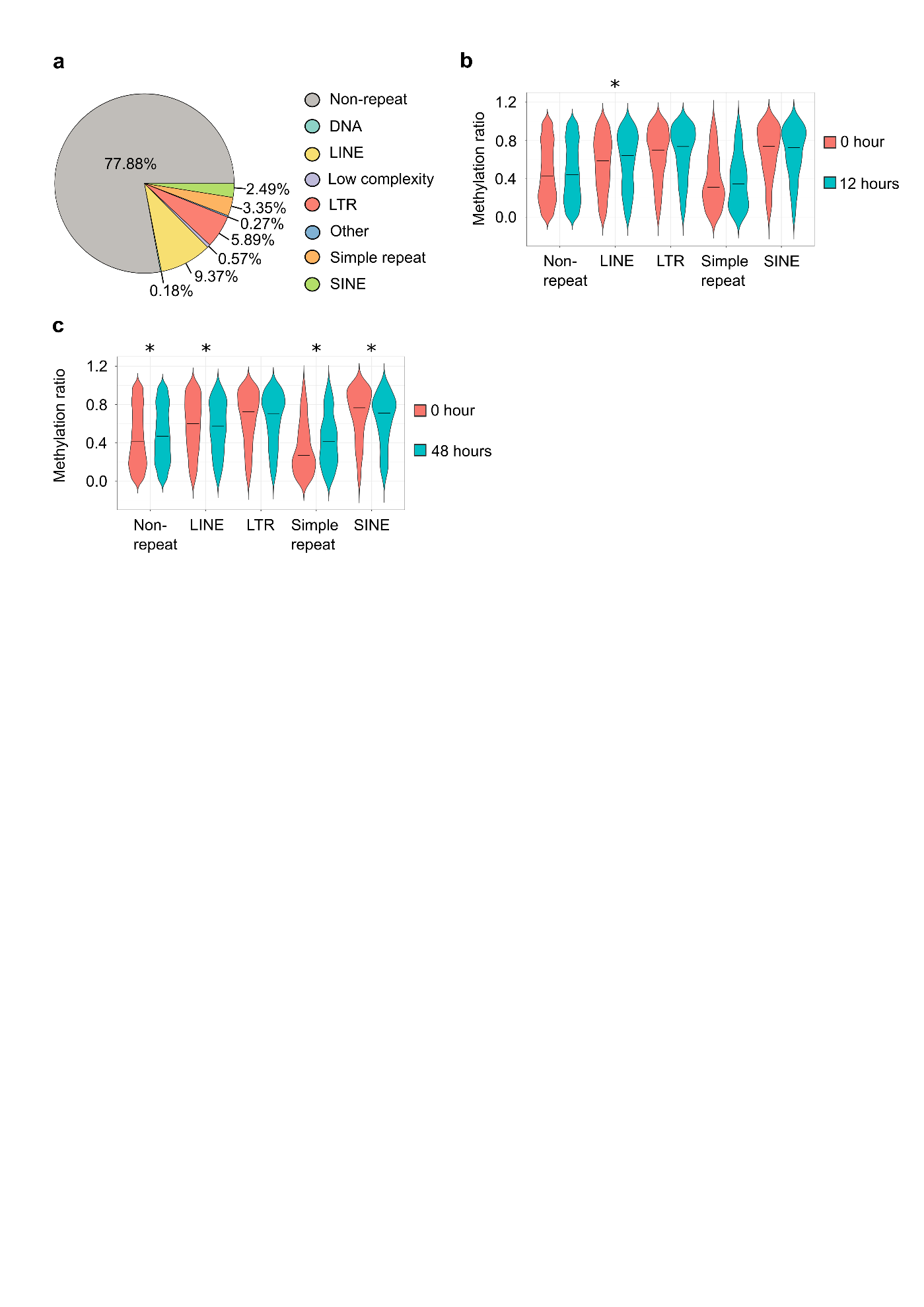


**Fig S4.** **Annotation of DMCs with non-repeats and repetitive elements.** **(a)** Pie plot annotating DMCs distributed within non-repeats and repetitive elements. **(b**-**c)** Methylation ratio of DMCs distributed within non-repeats and repetitive elements comparing **(b)** 0 and 12 hours and **(c)** 0 and 48 hours. 0 and 12 hours DMC distribution: Non-repeat n = 9351, LINE n = 1116, LTR n = 744, Simple repeat n = 414, SINE n = 299. 0 and 48 hours DMC distribution: Non-repeat n = 10920, LINE n = 1217, LTR n = 818, Simple repeat n = 483, SINE n = 324. Wilcoxon signed rank test was used to test for differences between 0 and 24 hours for each category. * significant at P < 0.05.


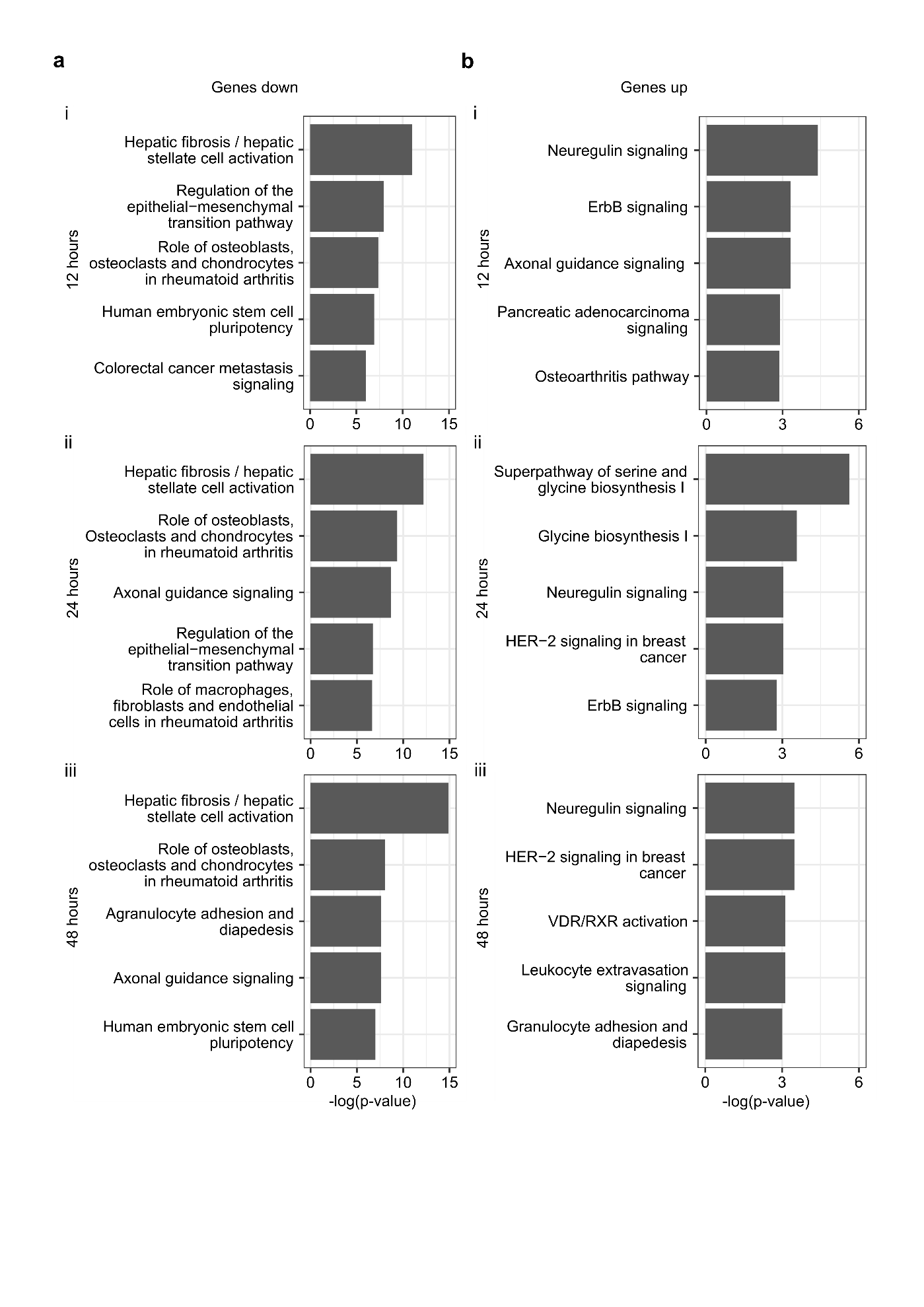


**Fig S5.** **Ingenuity Pathway Analysis (IPA) of RNA-seq genes.** (**ai-iii)** Top 5 pathways obtained from IPA of downregulated genes at **(i)** 12 hours, **(ii)** 24 hours and **(iii)** 48 hours. (**bi-iii)** Top 5 pathways obtained from IPA of upregulated genes at **(i)** 12 hours, **(ii)** 24 hours and **(iii)** 48 hours.


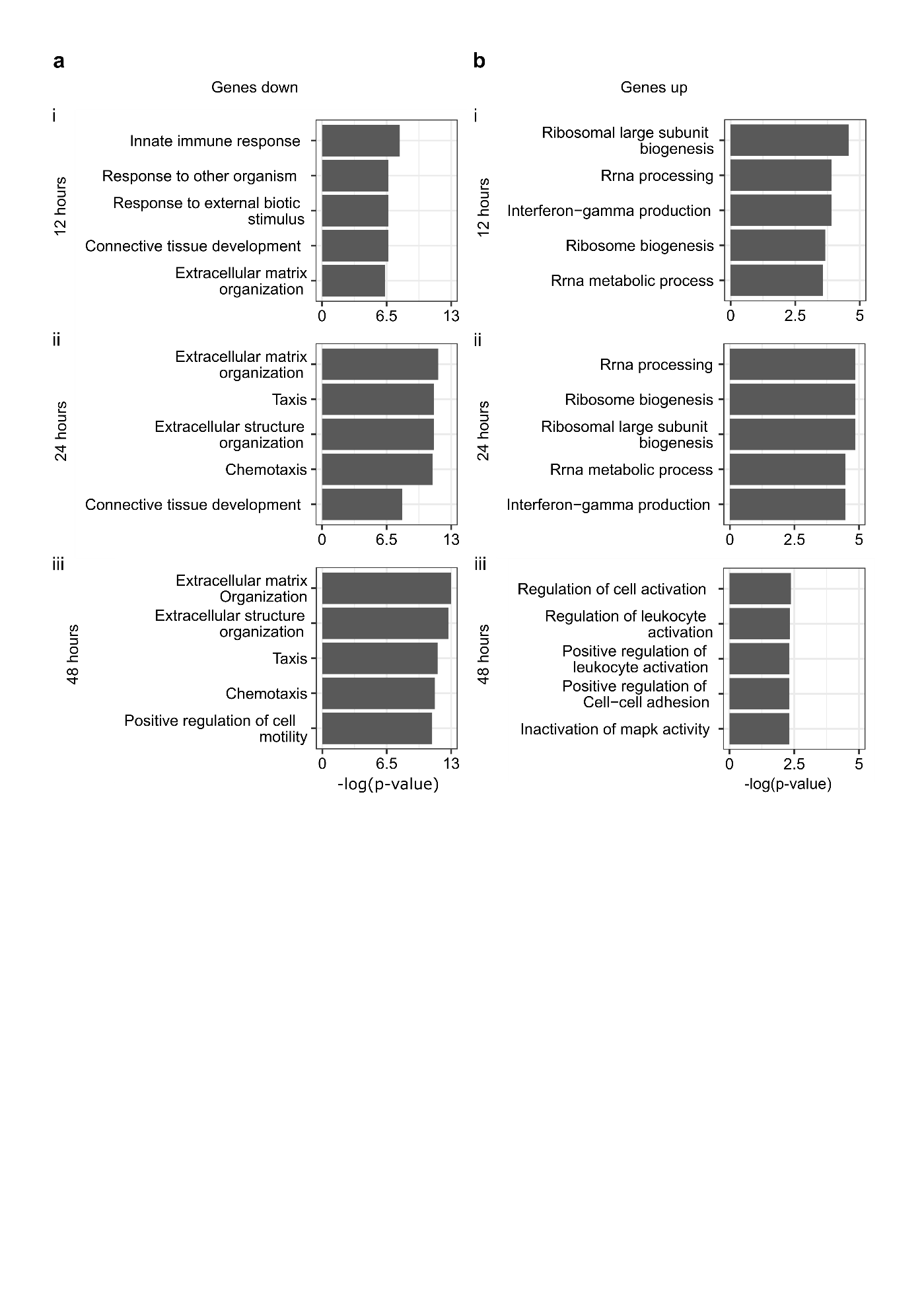


**Fig S6.** **Gene enrichment analysis of RNA-seq genes (ai-iii)** Top 5 pathways obtained from analysis of downregulated genes at **(i)** 12 hours, **(ii)** 24 hours and **(iii)** 48 hours. (**bi-iii)** Top 5 pathways obtained from analysis of upregulated genes at **(i)** 12 hours, **(ii)** 24 hours and **(iii)** 48 hours. All pathways were identified using clusterProfiler R Bioconductor package and Gene Ontology (GO) database.


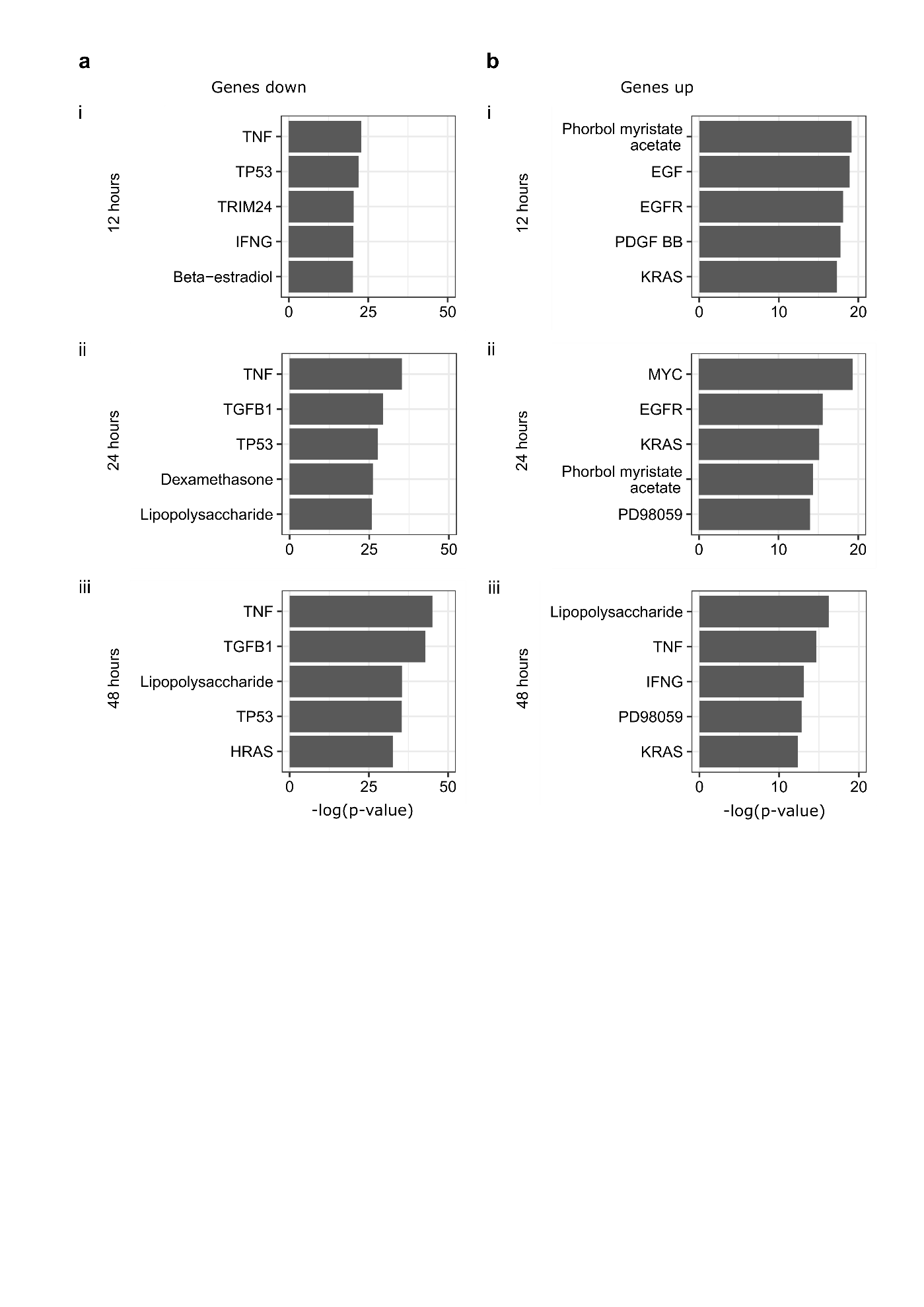


**Fig S7. Upstream regulatory analysis of RNA-seq genes conducted in IPA.** (**ai-iii)** Top 5 upstream regulators for downregulated genes at **(i)** 12 hours **(ii)** 24 hours and **(iii)** 48 hours. (**bi-iii)** Top 5 upstream regulators for upregulated genes at **(i)** 12 hours **(ii)** 24 hours and **(iii)** 48 hours.


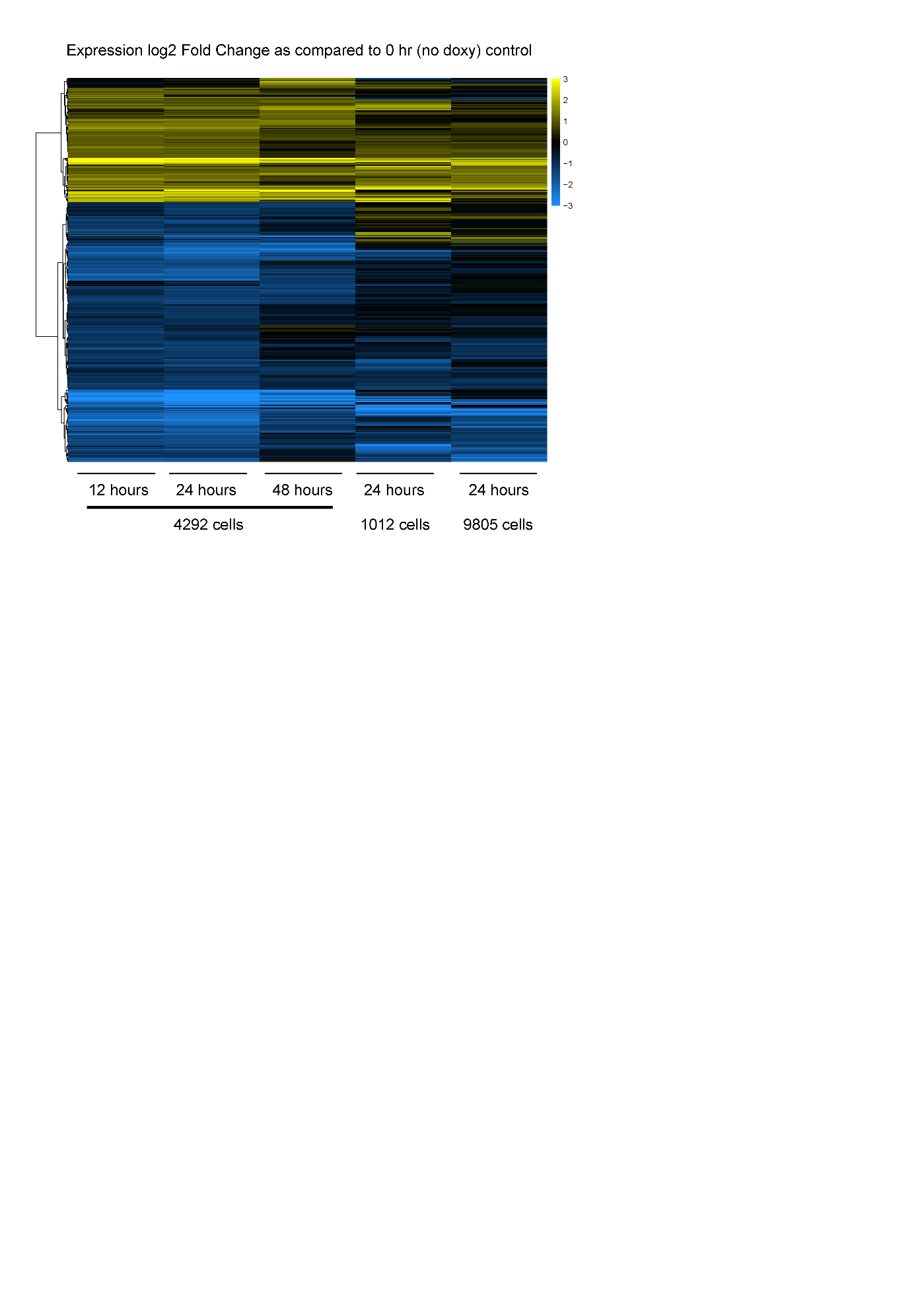


**Fig S8. RNA-seq analysis following oncogenic *Kras^G12D^* induction in 1012 and 9085 cell lines**. Log2 Fold change expression levels of 2091 DEGs identified in 4929 cells were normalized to the Z scale and plotted. Yellow: positive change. Blue: negative change. Black: no change.


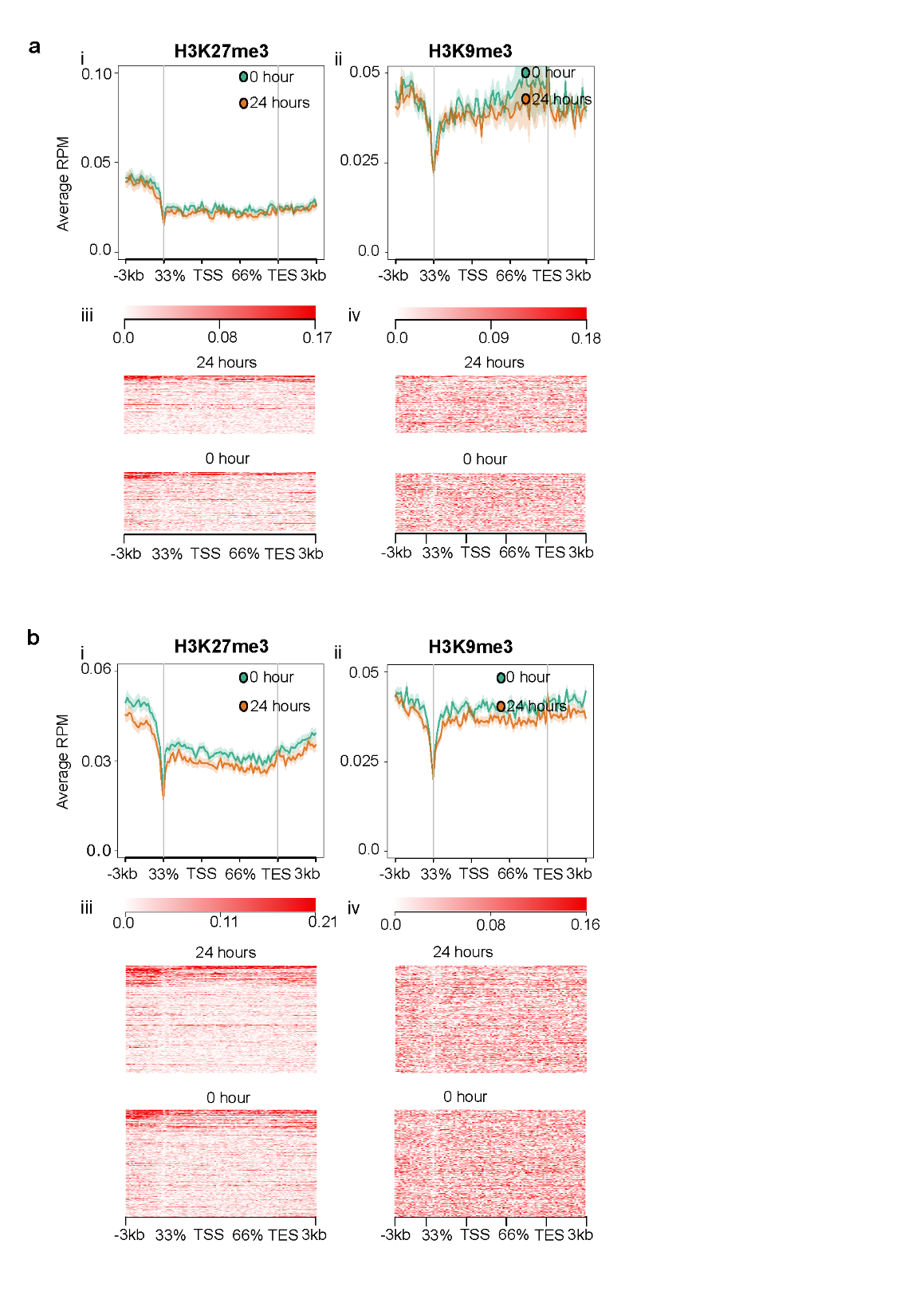


**Fig S9. Chromatin marks at gene bodies for up and downregulated transcripts following *Kras^G12D^* induction**. (**ai-ii)** Average profile plots of normalized H3K27me3 and H3K9me3 reads around the gene body for upregulated genes at 24 hours in the RNA-seq data (446 genes). Orange and green shaded area represent the standard error of the mean. (**aiii-iv)** Red heatmaps show normalized reads around the gene body for each upregulated gene. (**bi-ii)**Average profile plots of normalized H3K27me3 and H3K9me3 reads around the gene body for downregulated genes at 24 hours in the RNA-seq data (1165 genes). Color scheme is same as (**ai-ii)**. (**biii-iv)** Red heatmaps show normalized reads around the gene body for each downregulated gene.


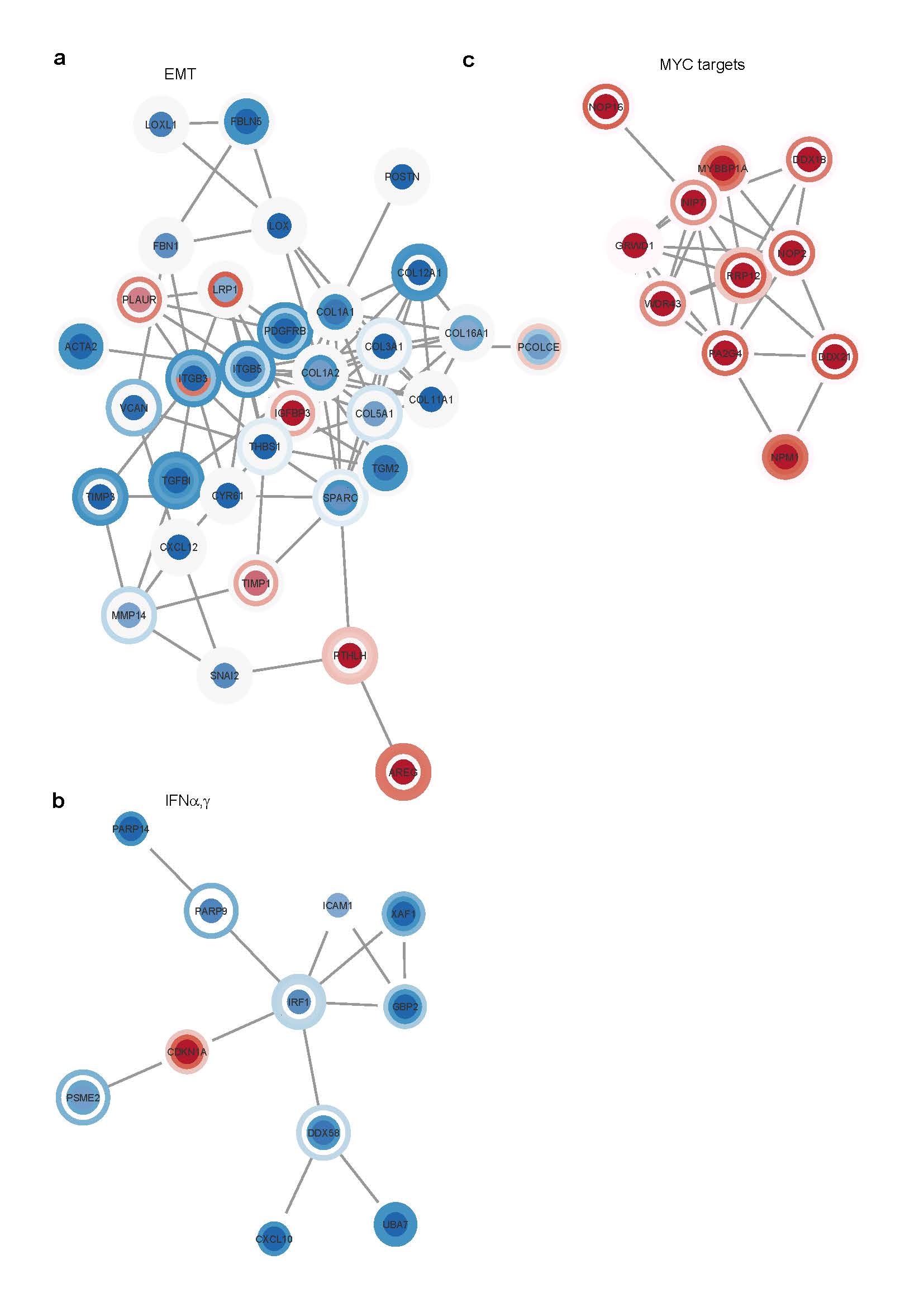


**Fig S10. Networks generated from genes associated with H3K27ac, H3K4me3 and H3K36me3 marks.** (**a)** EMT network. (**b)** IFN-α and γ network. (**c)** Myc targets network. Center red or blue dots represent upregulated and downregulated genes in the RNA-seq data set respectively. Concentric circles around the dots represent changes in H3K27ac, H3K4me3 and H3K36me3 respectively (going inside out) with red representing gain of the mark and blue representing loss of the mark. Dots are labeled with gene names in black.


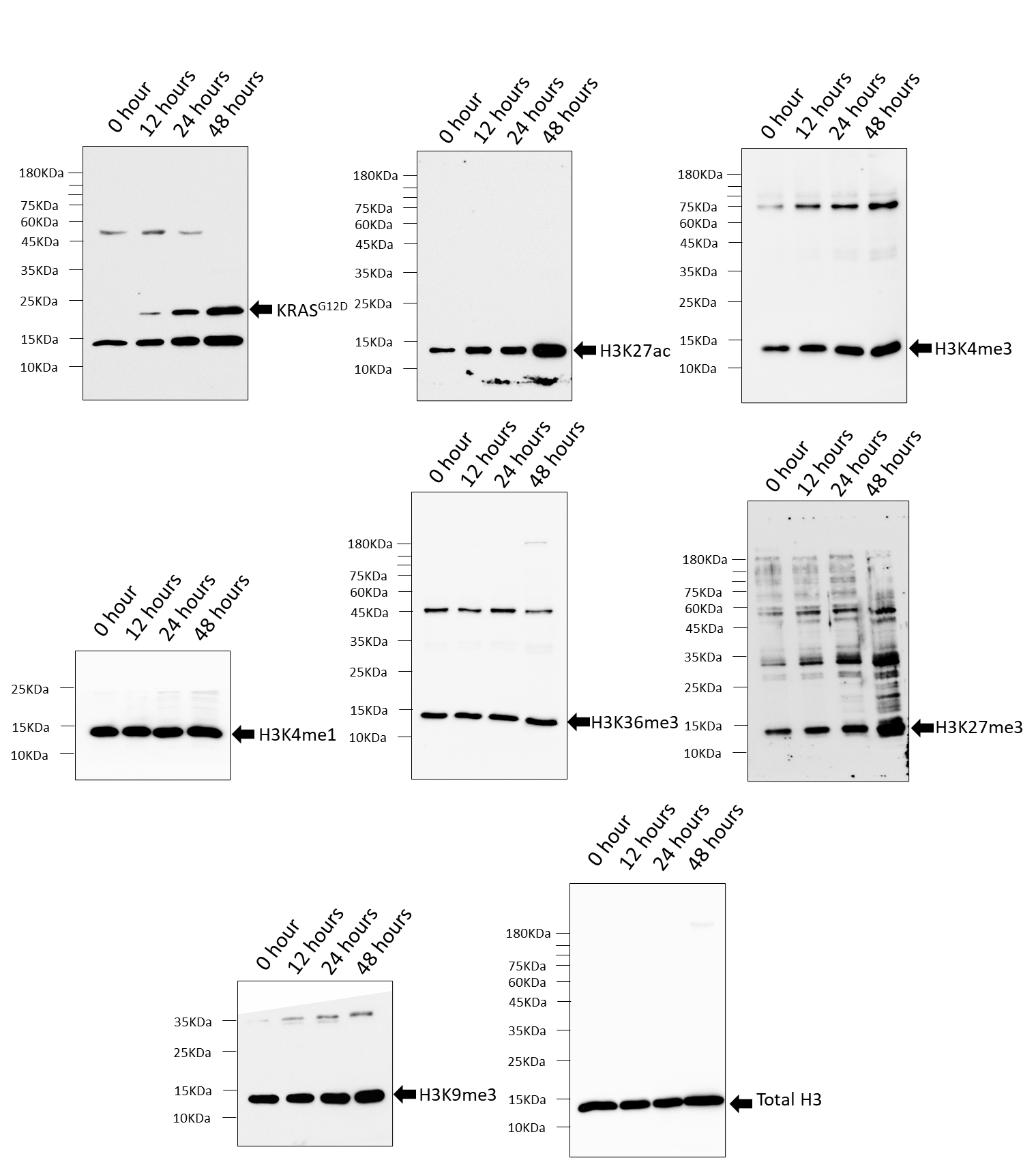


**Fig S11**. **Uncropped version of western blots present in Figure 1B of the main manuscript.** Western blot analysis was performed in 4292 i*Kra*s cell lines at 0, 12, 24 and 48 hours. Molecular weight is shown on the right and antibody target on the left of each blot.


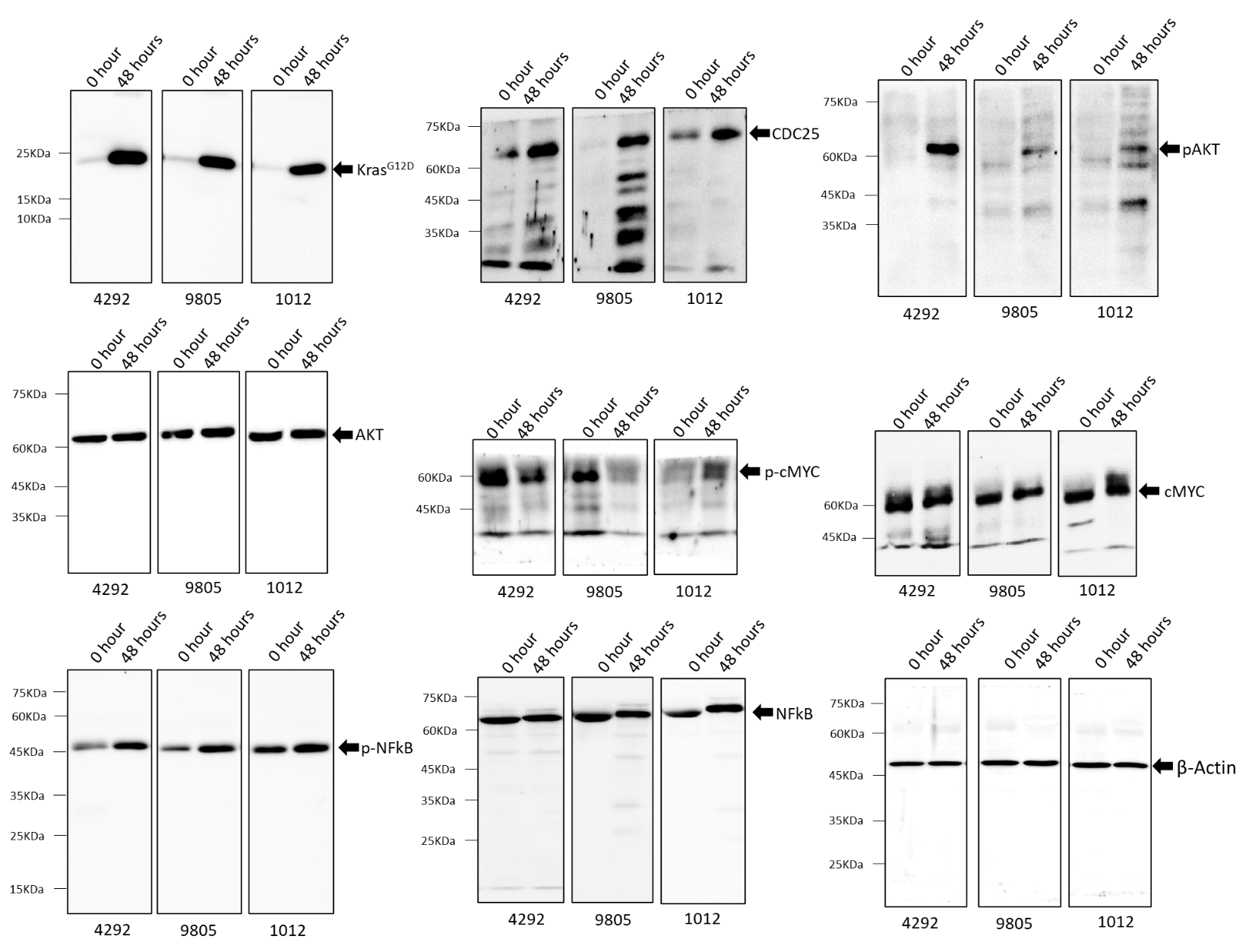


**Fig S12**. **Uncropped version of western blots present in Supplementary Figure 1.** Western blot analysis was performed in all i*Kra*s cell lines (4292, 9805 and 1012) at 0 and 48 hours. Molecular weight is shown on the right and antibody target on the left of each blot.


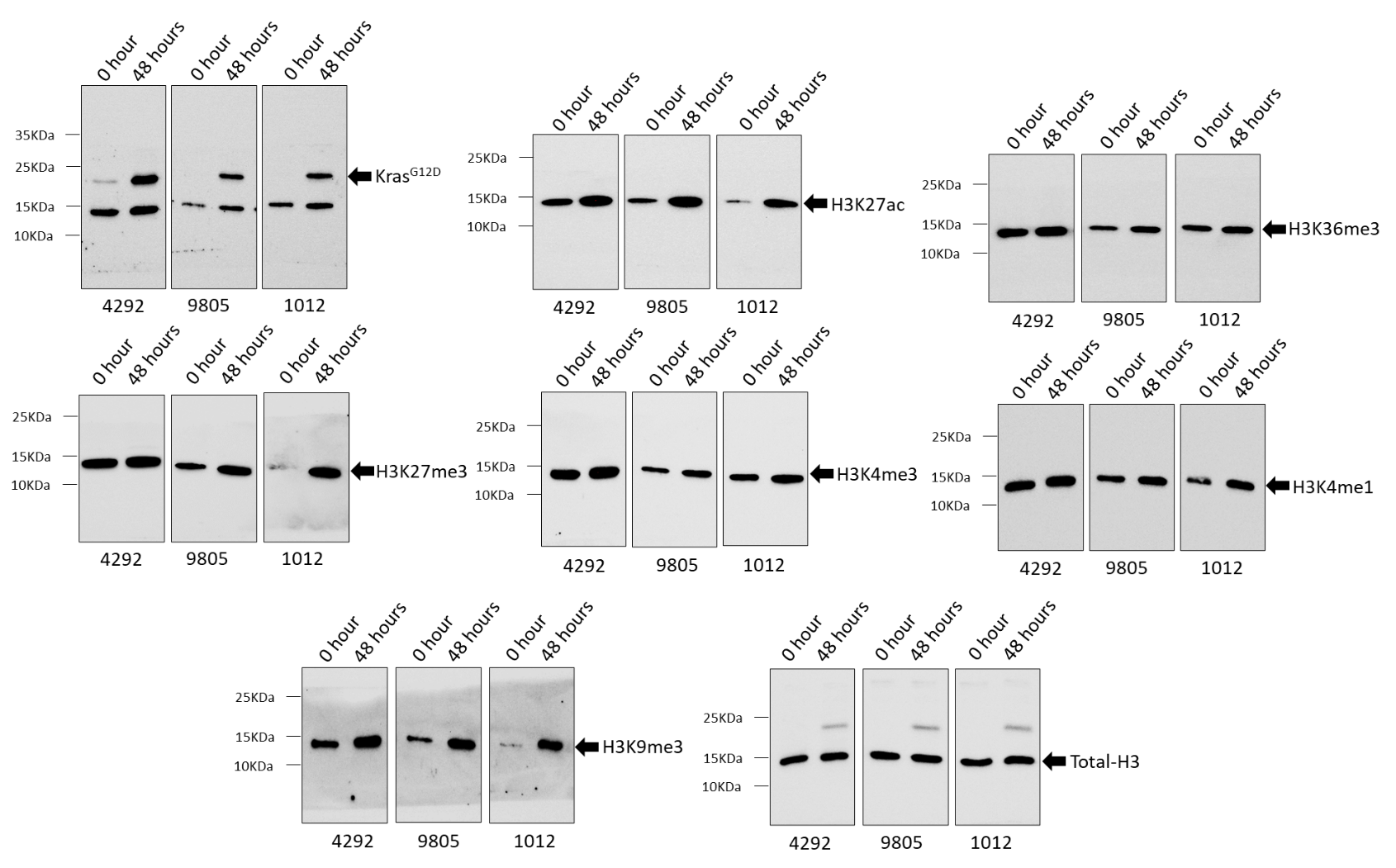


**Fig S13.** **Uncropped version of western blots present in Supplementary Figure 2.** Western blot analysis was performed in all i*Kra*s cell lines (4292, 9805 and 1012) at 0 and 48 hours. Molecular weight is shown on the right and antibody target on the left of each blot.
