# Supplementary material for: KrasG12D induces changes in chromatin territories that differentially impact early nuclear reprogramming in pancreatic cells
Source: Genome Biol. 2021 Oct 14;22:289. doi: 10.1186/s13059-021-02498-6 (PMC8518179; doi:10.1186/s13059-021-02498-6)
Supplement: Supplementary file 3 — Additional file 3. Review history. [file 13059_2021_2498_MOESM3_ESM.docx]

**Review history**

**First round of review**

**Reviewer 1**

This study by Kerketta et al provides a comprehensive genomics investigation of chromatin changes that occur downstream of KrasG12D in pancreatic cancer. This study is rigorous in its design and leads to several non-obvious conclusions that will be of high significance to the cancer epigenetics field. Because this study is comprehensive and rigorous in its design, i have no additional experiments to suggest to the authors. I support publication without delay.

**Reviewer 2**

Kerketta et al. perform a multi-omics integrative analysis of the effects of inducible mutant KRas expression in pancreas cancer cells generated from a genetic mouse model. They perform a series of superficial cell biology analyses, then go on to integrate data from ChIP-Seq, ATAC-Seq, RNA-Seq, and RBBS from one of the cell lines at variable time points. They conclude that mutant KRas induction results in heterochromatin reorganization and in the regulation of transcriptional programs driven through the activation (or repression) of super-enhancers and enhancers as well as promoters. Overall, the work is well performed although for some experiments it is not clear how many biological replicates were used; also, the integration with the transcriptional output is relatively superficial - as are the cell biology experiments reported at the beginning of the paper which - in my view - do not add much to the message of the manuscript. A major problem is that the paper is written as if the findings are pertinent to the effects of mutant KRas during pancreatic cancer initiation whereas - in fact - the authors use a series of cell lines generated from iKPC mice that harbour p53 inactivation and, therefore, the relevance of these findings to PDAC initiation is questionable. Obviously, the paper would greatly benefit from the analysis of the effects of doxycycline removal after induction, since this may be pertinent to the development of anti-RAS drugs. There are many overstatements (particuarly, in the first cell-biology part of the paper); the authors should carefully review the text to make sure that their statements are substantiated by the data. Finally, from a formal standpoint, many generic statements are repeated multiple times in the paper, the wording is a bit too verbose and the Results section would be streamlined to deliver a more clear-cut and concise message.

General comments
1. Re-write as appropriate taking into consideration the fact that they are using pancreatic cancer cells (e.g. last paragraph of page 15).
2. The issue of the mesenchymal-epithelial shifts mentioned in the text needs to be carefully revised based on comment 1 and also on the very loose definition of EMT and of acquisition of epithelial features. The data are not quite consistent: an epithelial shift is proposed in Figure 1 whereas an EMT is proposed to take place later in the paper (page 8 and subsequently), upon mutant KRas induction. For example:
    -Page 8, line 215, Page 9, line 216: In Figure 7d at 24hour iKras, EMT pathway is unchanged and TGF beta signaling is up. How does this fit with previous statement that "..cells transition from a.. mesenchymal state to a KrasG12D expressing (24 hrs) more epithelial-like one" ?
    -Page 9, line 221-222: How can it be explained that transcriptional regulators of differentiation are down-regulated in Kras expressing cells, when these cells are argued to be more epithelial than before dox induction of KrasG12D??
3. The multi-omics experiments are performed only in one cell line and at least some of the key findings should be validated in an independent line (not necessarily genome-wide, targeted validation should be enough).
4. A disclaimer should be included as to the possible effects of doxycycline on its own (probably not important, but best to mention this caveat).

Specific comments
1. Page 4, lines line 99-103: since they already have the DEG analysis in Supplementary table S1, they should include functional annotation analysis of the genes to determine whether a broader epithelial program is induced. The discrepancies with later findings and the fact that Cdh1 gene expression is gradually down-regulated in their RNAseq data speaks against acquisition of an epithelial phenotype. Immunostaining of typical epithelial markers in PDAC should be done so support this statement. Another point is to confirm their pathway enrichment analysis based on the PAPA array.
2. The size/numbers/expression of MAN- and nucleolin-positive cells should be quantified in control and iKras conditions from multiple images and cells.
3. Figure 2 deals with the changes in phospho-protein levels. The first point analyzed after addition of doxycycline in 12 h, which for signalling/phospho-protein analyses is quite late. This limitation should be considered in concluding about these experiments.
3. Page 6, line 164-165: the "...significant increase in the levels of all six histone marks in KrasG12D (+) cells at 48 hrs.." is not evident for the six histone marks in cell line 4292. E.g. in Figure 3d, H3K4me1 and H3K36me3 don't increase or change slightly. On which basis are the increases significant? Also, in Supplementary Figure 1 in cell line 4292, are the small increases (if any?) of H3K4me1 and H3K36me3 significant? Therefore, the authors either need to quantify all histone mark levels in several experiments at least in 4292 line to test if changes are really significant, or down-play thes tatement.
4. Page 7, line 166-168: For K9me3 in Figure 4f, it doesn't look like it moves to the nuclear periphery but rather an increase of intensity and increase in number of positive nuclei. K27me3 in Figure 4e is already in the nuclear periphery in controls. There could be some mislabeling in the panels in Figure 4 as some of the shown histone mark stainings do not correspond to description in text. Either way: The mentioned move of K9me3 and K27me3 to the nuclear periphery and the global increase in other marks should be quantified in control and iKras conditions from multiple images and nuclei (K27me3 and K9me3 are in Figure 4e and 4f). In addition, the authors should consider whether the changes in total H3 and/or histone marks reflect differences in cell cycle distribution (which would lead to doubling of total histones).
5. Page 7, line 176: How many regions gain and how many regions lose accessibility? The sentence "These analyses revealed 6127 regions that gain and 559 regions that change accessibility.."  does not make sense. Is it 6127 gain and 559 loss regions?
6. Page 7, line 179: In methods it says: "The identified regions were annotated with genes if they were located within ± 10kb of gene transcription start sites (TSSs)". The authors should provide numbers for how many of the gained regions and how many of the lost regions that were used for annotation to genes given the relatively narrow window around TSS they use (+/-10kb).
7. Page 7, line 179-181: The authors claim that global chromatin accessibility increased the most around TSS areas, but yet they only analyze 3kb around TSS. The authors either need to compare the increase here with changes in other genomic regions they defined earlier or modify this sentence.
8. Page 7, line 182-186: Authors need to link the expression changes of the genes within newly open chromatin regions. Are the genes that map to the mentioned pathways upregulated? Is there a correlation between differential chromatin accessibility and gene expression changes? This is an obvious question that is not addressed at all.
9. Page 9, line 225-227: Super-enhancer (SE)-associated genes are normally expressed at high levels (e.g. PMID: 23582322 and PMID: 23582323), in fact at higher levels than genes associated with normal enhancers. How can the authors explain that most of the TF genes associated with SEs at 24h in Figure 7f are down-regulated as compares to time 0?
10. Page 12, line 303: As H4K4me1 also marks enhancers, the authors should analyze H3K4me1 in more distal regions from TSS of UP and DOWN genes (within the threshold of 10kb as the authors has set).


Minor points
1. Page 5, line 116: MYC gene expression is upregulated in each time point in iKras conditions in the RNAseq data. In figure 2d it is indicated with a blue center circle which according to figure legend indicates down-regulated protein?
2. Page 5, line 129: I tink that the terms "activating phosphorylation" and "inhibitory phosphorylation" are not used properly here. Please revise.
3. Page 5, line 127-128: In Figure 2d according to figure legend, outer concentric circle around MYC indicates inhibiting protein activity as the color is light blue.
4. Page 6, line 162: H3K27ac also marks enhancers and H3K4me1 also marks enhancers.
5. What is an "oncogenic cell nucleus"?
6. I do not think that the ultrastructural images add anything to the paper unless there is appropriate image quantification.
7. Page 7, line 189: "ChIP-Seq for H3K27ac revealed an overall gain in enrichment of differentially bound (DB) regions". Bound by what? I would say differentially marked regions.
8. Page 8, line 199: There should be a short paragraph explaining that non-promoter regions associate with nuclear periphery and reference for this.
9. Page 8, line 207: The time-point dependent signal intensity variation is not shown in Figure 7c. Where is this shown?

**Authors’ response to reviewers**

# General comments

**Comment 1.** Re-write as appropriate taking into consideration the fact that they are using pancreatic cancer cells (e.g. last paragraph of page 15).

***Response 1:*** *Revisions of the results sections has eliminated the need for this discussion paragraph regarding the nuclear shape and structure, thus is has been removed.*

**Comment 2**. The issue of the mesenchymal-epithelial shifts mentioned in the text needs to be carefully revised based on comment 1 and also on the very loose definition of EMT and of acquisition of epithelial features. The data are not quite consistent: an epithelial shift is proposed in Figure 1 whereas an EMT is proposed to take place later in the paper (page 8 and subsequently), upon mutant KRas induction.

***Response 2****: We agree with the reviewer and have adjusted statements about EMT in the results section accordingly.*

**Comment 2b:**-Page 8, line 215, Page 9, line 216: In Figure 7d at 24hour iKras, EMT pathway is unchanged and TGF beta signaling is up. How does this fit with previous statement that "..cells transition from a.. mesenchymal state to a KrasG12D expressing (24 hrs) more epithelial-like one" ?

***Response 2b:*** *We agree with the reviewer, in this instance there is not sufficient change in the super-enhancer elements to make the statement. Thus the sentence has been removed.*

**Comment 2c**-Page 9, line 221-222: How can it be explained that transcriptional regulators of differentiation are down-regulated in Kras expressing cells, when these cells are argued to be more epithelial than before dox induction of KrasG12D??

***Response 2c****: We clarified that our work identifies transcription factors involved in the Kras pathway. Many of these transcription factors play pleotropic roles in cell proliferation, differentiation, migration, senescence, apoptosis, autophagy, etc. For this reason, we have not assigned in our manuscript an exclusive role to any of them. We are optimistic that future, careful functional studies, which are beyond the scope of the current manuscript, will further elucidate their specific role.*

**Comment 3**. The multi-omics experiments are performed only in one cell line and at least some of the key findings should be validated in an independent line (not necessarily genome-wide, targeted validation should be enough).

***Response 3****: The authors agree with the reviewer that a single cell line may not allow for the data to have greater translation in the areas of genomic and epigenomic alterations.*

*However, authors note that the origin of the cells, the conditions of culture, additional genomic variants may also play a huge role in the establishment and maintenance of transcriptomic and epigenetic alterations. Thus, we have validated key findings in the independently generated 1012 and 9805 cell lines derived from the same mouse background. As the reviewer noted, these animals harbor p53 inactivation which may have a role in establishing the epigenetic landscape that we show is altered upon Kras induction.*

*PAPA genes were validated and are described in Supplementary Figure 1*

LINES 149-152: “As a control, western blots confirm the upregulation of phosphorylated AKT1, CDC25C, and NF-κB and downregulation of inhibitory MYC phosphorylation in the 4292, 9805 and 1012 *Kras^G12D^* cell lines at 48 hrs (Supplementary Figure 1).”

*Transcriptional changes of the 2091 DEG are confirmed and shown in Supplementary Figure 8 and Supplementary Table 1*

LINES 298-303: “To confirm the gene expression patterns and consistency of alterations in key pathways, transcriptomics were completed on the 1012 and 9805 cell lines (Supplementary Table 1). Filtered to match the set of 2091 genes altered by the 4292 cells, we observe that approximately 85% of the time the direction of the DEG was matched across all 3 cell lines. Additionally, genes of the IFN-alpha, IFN-gamma, EMT, Myc, Mtorc1, and Kras up/down regulation were similarly altered in these three *Kras^G12D^* inducible cell lines.”

*ChIP H3K27ac and H3K4me1 targets are confirmed and shown in supplementary figure 3* LINES 203-208: “An independent ChIP assay performed on all three *Kras^G12D^* cell lines (4292, 9805 and 1012) in the promoter region of *Btc, Etv4, Cdkn1a* and *Npm1* showed an increase in the deposition of H3K27ac and H3K4me3 marks and a decrease in the deposition of those same marks at *Itgb5, Pdgfrb, Wnt10b* and *Uba7*. A confirmation that *Kras^G12D^* induction leads to the same remodeling of activating chromatin marks as seen on ChIP-seq analysis (Supplementary Figure 3).”

**Comment 4**. A disclaimer should be included as to the possible effects of doxycycline on its own (probably not important, but best to mention this caveat).

***Response 4****: Have added the following sentence to the materials and methods.*

LINES 458-460: “The inducible *Kras^G12D^* expression system utilized here does not allow for the elimination of any confounding effects of that doxycycline treatment alone may initiate on the transcriptome and epigenome of these cells.”

# Specific comments

**Comment 1**. Page 4, lines line 99-103: since they already have the DEG analysis in Supplementary table S1, they should include functional annotation analysis of the genes to determine whether a broader epithelial program is induced. The discrepancies with later findings and the fact that Cdh1 gene expression is gradually down-regulated in their RNAseq data speaks against acquisition of an epithelial phenotype. Immunostaining of typical epithelial markers in PDAC should be done so support this statement. Another point is to confirm their pathway enrichment analysis based on the PAPA array.

***Response 1****: As per the DEG analysis, Supplementary table 1 has been reduced to only include genes that meet the filter thresholds (expression fold change ≥ |2| and FDR ≤ 0.05) for RNASeq in at least one of the 3 timepoints (12hr, 24hr, 48hr). Functional annotations of those 2091 genes, as they align with the pathway enrichment analysis of Figure 7 have also been added. We agree that these important pathway analysis aid in understanding of the significantly up and downregulated pathways discussed throughout the manuscript.*

*Considering the EMT pathway, reviewer will note the abundance of downregulation in the genes associated with that pathway, supporting the epithelial markers seen by western blot in these cells. Regarding Cdh1, it does not meet cut off values for DEG and is no longer listed in Supplemental Table 1.*

*Additional western blots were completed to confirm the PAPA arrays, that match pathways reflected in the RNAseq data. This data is presented as part of Supplementary Figure 1.*

**Comment 2**. The size/numbers/expression of MAN- and nucleolin-positive cells should be quantified in control and iKras conditions from multiple images and cells.

***Response 2****: As per reviewer suggestions, the superficial treatment of cell biology experiments do not add much to the message of the manuscript, which focuses on the epigenetic alterations. Thus, the cell staining experiments have been removed from the manuscript.*

**Comment 3**. Figure 2 deals with the changes in phospho-protein levels. The first point analyzed after addition of doxycycline in 12 h, which for signalling/phospho-protein analyses is quite late. This limitation should be considered in concluding about these experiments.

***Response 3****: We agree that some early phosphorylation events may be lost by the first PAPA arrays being tested at 12hr. However, we do note that protein levels of KrasG12D continue to increase over the 12 and 24hr timepoints and thus may be initiating additional and novel pathways. We added the following sentence to describe this limitation.*

LINES 113-116: *“Kras^G12D^* induction by western blot (Figure 1c) shows moderate expression and levels increase out to 24 hrs. By mirroring these timepoints in the PAPA array, we may miss early signaling events, but we can observe phosphorylation changes that are initiated at 12 hrs and maintained by *Kras^G12D^* in these cells.”

**Comment 3**. Page 6, line 164-165: the "...significant increase in the levels of all six histone marks in KrasG12D (+) cells at 48 hrs.." is not evident for the six histone marks in cell line 4292.

E.g. in Figure 3d, H3K4me1 and H3K36me3 don't increase or change slightly. On which basis are the increases significant? Also, in Supplementary Figure 1 in cell line 4292, are the small increases (if any?) of H3K4me1 and H3K36me3 significant? Therefore, the authors either need to quantify all histone mark levels in several experiments at least in 4292 line to test if changes are really significant, or down-play thes tatement.

***Response 3****: Authors thank the reviewer for this important comment. Western blots have been quantified by densitometry and values are provided in text and as Figure 1c.*

**Comment 4**. Page 7, line 166-168: For K9me3 in Figure 4f, it doesn't look like it moves to the nuclear periphery but rather an increase of intensity and increase in number of positive nuclei. K27me3 in Figure 4e is already in the nuclear periphery in controls. There could be some mislabeling in the panels in Figure 4 as some of the shown histone mark stainings do not correspond to description in text. Either way: The mentioned move of K9me3 and K27me3 to the nuclear periphery and the global increase in other marks should be quantified in control and iKras conditions from multiple images and nuclei (K27me3 and K9me3 are in Figure 4e and 4f). In addition, the authors should consider whether the changes in total H3 and/or histone marks reflect differences in cell cycle distribution (which would lead to doubling of total histones).

***Response 4****: The reviewer astutely brings to light the fact that the cellular data in this manuscript are more complex than the limited figures could convey. Similarly, the strength of this manuscript is the epigenetic changes not the cellular biology. Fully describing the 3D alterations and corresponding interplay with cell cycle distribution are beyond the scope of this manuscript. Thus, Figures 1, 3, and 4, have been modified or removed.*

**Comment 5**. Page 7, line 176: How many regions gain and how many regions lose accessibility? The sentence "These analyses revealed 6127 regions that gain and 559 regions that change accessibility.." does not make sense. Is it 6127 gain and 559 loss regions?

***Response 5****: Confirmed that the statement should be modified as reviewer suggested. Numbers have changed due to expanded ±10kb window for the regions around TSS. Thus, sentence has been revised to:*

LINES 159-160: “These analyses revealed 6123 regions that gain and 559 regions that lost accessibility (FDR ≤ 0.05; Figure 3ai).”

**Comment 6**. Page 7, line 179: In methods it says: "The identified regions were annotated with genes if they were located within ± 10kb of gene transcription start sites (TSSs)". The authors should provide numbers for how many of the gained regions and how many of the lost regions that were used for annotation to genes given the relatively narrow window around TSS they use (+/-10kb).

***Response 6****: Adjusted sentences to clarify the types of regions that were gained or lost in accessibility.*

LINES : “Most regions that change accessibility were in introns (33%), promoters (within 10kb, 29%) and intergenic (29%) (Figure 3aiii). Hence, a characteristic of the *Kras^G12D^* response is predominantly a gain in accessible chromatin, affecting 2711 genes (2564 in their introns, 1563 in promoters, from 3595 and 1830 peaks, respectively), while only 323 genes became inaccessible (312 in their introns, 79 in promoters, from 346 and 82 peaks, respectively; Figure 3aii).”

**Comment 7**. Page 7, line 179-181: The authors claim that global chromatin accessibility increased the most around TSS areas, but yet they only analyze 3kb around TSS. The authors either need to compare the increase here with changes in other genomic regions they defined earlier or modify this sentence.

***Response 7****: Modified sentence to clarify and updated figures 3aii and iii, 3bi and ii*

LINES : “In fact, when considering the ±10kb region surrounding gene transcription start sites (TSS), the global chromatin accessibility in *Kras^G12D^* induced cells increased the most in areas adjacent to the TSS (7.7% increase in the -1kb promoter region and 10.4% in first intron) (Figure 3bi-ii).”

**Comment 8**. Page 7, line 182-186: Authors need to link the expression changes of the genes within newly open chromatin regions. Are the genes that map to the mentioned pathways upregulated? Is there a correlation between differential chromatin accessibility and gene expression changes? This is an obvious question that is not addressed at all.

***Response 8****: The authors agree that the integration of the expression and epigenetic data is an important to consider. Authors have included two gene examples in Figure 3d. Integration of the accessibility, chromatin marks, methylation and transcriptomics was reserved for the final figure, figure 9. Here we see that the open chromatin (red) does consistently correlate with the increased expression of many genes.*

**Comment 9**. Page 9, line 225-227: Super-enhancer (SE)-associated genes are normally expressed at high levels (e.g. PMID: 23582322 and PMID: 23582323), in fact at higher levels than genes associated with normal enhancers. How can the authors explain that most of the TF genes associated with SEs at 24h in Figure 7f are down-regulated as compares to time 0?

***Response 9****: The expression levels for all genes and transcription factors have been modified as figures 5 ei-ii and fi-ii. Previous figures included transcription factors that were changing in both the 0 and 24h groups and have subsequently been removed. As the identification of super-enhancers by the ROSE algorithm do not incorporate the 3D structure of the nucleus, not all transcriptional regulation can solely be described by the H3K27ac*

*super-enhancer mark. Thus, sentences were added to indicate that ongoing studies in 3D will be important to better understand the 3D regulation of these enhancers.*

LINES 337-339: “Mapping the ChromHMM states along a 2D linear gene representation depicts how chromatin states alternate along segments of DNA, although they likely represent contacts that these genes make with chromatin proteins which aggregate in distinct 3D domains (66).”

LINES 227-229: “Ongoing studies that consider the impact of three-dimensional chromatin structure and regulation of super-enhancers on more distal genes will elucidate additional levels of regulation that may explain differences from the simple model that super-enhancers always up-regulate cis-acting genes.”

LINES 214-217: “Generally, genes associated with super-enhancers have higher expression compared to the same genes when the super-enhancers were not formed. This consistent change in gene expression supports a clear functional role for these changes in super- enhancer architecture, downstream of oncogene induction.”

**Comment 10**. Page 12, line 303: As H4K4me1 also marks enhancers, the authors should analyze H3K4me1 in more distal regions from TSS of UP and DOWN genes (within the threshold of 10kb as the authors has set).

***Response 10****: We agree with the reviewer given the consideration of H3K4me1 as an enhancer, thus we have widened the window of observation and it is now set at -10kb to*

*+10kb and included this as a set of panels in Figure 8a,b.*

# Minor points

**Comment 1**. Page 5, line 116: MYC gene expression is upregulated in each time point in iKras conditions in the RNAseq data. In figure 2d it is indicated with a blue center circle which according to figure legend indicates down-regulated protein?

***Response 1****: The reviewer wisely points out the need for clarification in these disconnected statements. Thus, we have modified Supplementary Table 1 to include only those genes that match our cut off values. MYC gene expression had a log2 fold change of 0.7, 0.4 and 0.4 in the RNAseq thus falling below the log2 fold change cut off of 1 (fold change = 2) that is set by our analysis. These transcriptional changes are very small and thus may be difficult to correlate with activity and protein phosphorylation events. For the PAPA arrays, the MYC phosphorylation has been normalized to unphosphorylated MYC levels and fold change calculated for the phosphorylation events. Both pSer62 and pThr58 are downregulated at 12, 24, and 48hrs (supplementary table 2) and thus colored with a blue inner circle. Modifications to Figure 2d legend and text to specify this.*

**Comment 2**. Page 5, line 129: I tink that the terms "activating phosphorylation" and "inhibitory phosphorylation" are not used properly here. Please revise.

***Response 2****: We have reworded the MYC and RB1 phosphorylation information to increase clarity.*

LINES 132-138: “Congruently, 24 hrs after *Kras^G12D^* induction, we noted activation of early response genes products, such as ELK-1 (pSer383, fold change = 1.6) and JUN (pSer73, fold change = 2.46), as well as activation of immediate early genes like MYC by downregulation of inhibitory phosphorylation (pSer62, fold change = 0.4, pThr58, fold change = 0.6) (Supplementary Table 2 and Figure 2d, bold letters) (7-11). Additionally, downstream of MYC, phosphorylation of RB1 (pSer780, fold change = 1.8), (Supplementary Table 2 and Figure 2 d, bold letters) releases E2F to mediate cell cycle progression and DNA synthesis(12,13).”

**Comment 3**. Page 5, line 127-128: In Figure 2d according to figure legend, outer concentric circle around MYC indicates inhibiting protein activity as the color is light blue.

***Response 3****: We have modified description of MYC, as indicated in previous comment, to add clarity as the downregulation of an inhibitory phosphorylation would give rise to increased activation of the gene. Similar statements are added in results and the Figure 2d description:* LINES 120-126: “We then categorized the protein phosphorylation status using two criteria, for each time point, based on their functional effect:1. whether the magnitude of phosphorylation increased or decreased and 2. whether they are known to be activating or inhibiting modifications. Phosphorylation states were categorized as “activity down” if either an activating protein phosphorylation decreased in magnitude, or an inhibitory phosphorylation increased (Figure 2c-d, Supplementary Table 2). Phosphorylation states were categorized as “activity up” if an activating phosphorylation increased in magnitude, or an inhibitory phosphorylation decreased.”

Figure 2 Legend: “We interpret the effect on activity by the combination of the mark’s effect and its level. For instance, an inhibitory mark decreasing should lead to a more active protein. Some proteins have both inhibitory and activating phosphorylations, hence, will have multiple colors in the outer concentric circle.”

**Comment 4**. Page 6, line 162: H3K27ac also marks enhancers and H3K4me1 also marks enhancers.

***Response 4****: We have added these labels as requested.*

**Comment 5**. What is an "oncogenic cell nucleus"?

***Response 5****: As the cellular staining and nuclear structure figures were removed, this comment was likewise removed as these features are no longer described.*

**Comment 6**. I do not think that the ultrastructural images add anything to the paper unless there is appropriate image quantification.

***Response 6****: As per comments of reviewer previously, cellular studies and staining added little to the epigenetic focus of the manuscript and were therefore removed.*

**Comment 7**. Page 7, line 189: "ChIP-Seq for H3K27ac revealed an overall gain in enrichment of differentially bound (DB) regions". Bound by what? I would say differentially marked regions.

***Response 7****: Authors agree with the suggestion of the reviewer and have re-written as differentially marked regions.*

**Comment 8**. Page 8, line 199: There should be a short paragraph explaining that non-promoter regions associate with nuclear periphery and reference for this.

***Response 8****: As cellular studies were removed, sentence was modified as follows with references added.*

LINES 186-189: “Next, we studied marks that associate with inactive chromatin. We found that the facultative heterochromatin mark, H3K27me3, locates primarily outside of promoters in intergenic regions upon *Kras^G12D^* expression (Figure 6eiii), which is congruent with cell biology data (25-27) that show accumulation of non-promoter regions and heterochromatin at the nuclear periphery and LADs.”

**Comment 9**. Page 8, line 207: The time-point dependent signal intensity variation is not shown in Figure 7c. Where is this shown?

***Response 9****: Supplemental table 3 was added to include the signal intensity values for super-enhancers unique to each timepoint and those overlapping between the 0h and 24h timepoints.*

**Second round of review**

**Reviewer 2**

The authors have responded to most of my requests adequately. They have removed from the ms. the cell biology-related component which was rather weak. However, there are a few places in the ms. where this change has not been reflected (in the Introduction, at the beginning of the discussion, etc). Please check carefully for this.
Otherwise, I think that the manuscript has improved considerably.

**Authors’ response to reviewers**

Reviewer #2: The authors have responded to most of my requests adequately. They have removed from the ms. the cell biology-related component which was rather weak. However, there are a few places in the ms. where this change has not been reflected (in the Introduction, at the beginning of the discussion, etc). Please check carefully for this. Otherwise, I think that the manuscript has improved considerably.

*We thank Reviewer #2 again for the astute observations and requests for revisions. We agree that the changes have improved manuscript considerably and clarified much of the writing. We have read through introduction and discussion again and removed the residual items with reference to cell biological data that has been removed.*
